# Comprehensive Pan-Cancer Analysis of the Prognostic and Immunological Roles of the METTL3/lncRNA-SNHG1/miRNA-140-3p/UBE2C Axis

**DOI:** 10.3389/fcell.2021.765772

**Published:** 2021-11-10

**Authors:** Xiulin Jiang, Yixiao Yuan, Lin Tang, Juan Wang, Qianqian Liu, Xiaolan Zou, Lincan Duan

**Affiliations:** ^1^ Key Laboratory of Animal Models and Human Disease Mechanisms of Chinese Academy of Sciences and Yunnan Province, Kunming Institute of Zoology, Kunming, China; ^2^ Department of Thoracic Surgery, The Third Affiliated Hospital of Kunming Medical University, Kunming, China

**Keywords:** epigenetics, SNHG1, miRNA-140-3p, UBE2C, pan-cancer, prognosis, immunotherapy, drug sensitivity

## Abstract

Growing evidence has demonstrated that UBE2C plays a critical role in cancer progression, but there is no study focusing on the prognosis, upstream regulation mechanism, and immunological roles of UBE2C across diverse tumor types. In this study, we found that UBE2C was elevated in this human pan-cancer analysis, and high expression of UBE2C was correlated with poor prognosis. In addition, UBE2C expression was markedly associated with tumor mutation burden (TMB), microsatellite instability (MSI), immune cell infiltration, and diverse drug sensitivities. Finally, we showed that the METTL3/SNHG1/miRNA-140-3p axis could potentially regulate UBE2C expression. N(6)-Methyladenosine (m6A) modifications improved the stability of methylated SNHG1 transcripts by decreasing the rate of RNA degradation, which lead to upregulation of SNHG1 in non-small cell lung cancer (NSCLC). *In vitro* functional experiments showed that SNHG1, as a competing endogenous RNA, sponges miR-140-3p to increase UBE2C expression in NSCLC cell lines. Our study elucidates the clinical importance and regulatory mechanism of the METTL3/SNHG1/miRNA-140-3p/UBE2C axis in NSCLC and provides a prognostic indicator, as well as a promising therapeutic target for patients with NSCLC.

## Introduction

Cancer has become one of the major reasons for cause of death in the world, resulting in great social and economic burden. Although there are great improvements in the diagnosis and treatment of cancer, the cure rate for cancer remains low (Siegel et al., 2019). Therefore, it seems extremely significant for identification of the specificity and sensitivity biomarkers for diagnosis and treatment of cancer.

As a conjugating enzyme, ubiquitin-conjugating enzyme 2C (UBE2C) plays an indispensable role in cancer progression (Hao et al., 2012). It has been shown that UBE2C is mainly involved in the mitotic spindle control through antagonism of the function of USP44 (Stegmeier et al., 2007).

Mounting evidence has shown that UBE2C was upregulated in cancers such as brain cancer (Donato et al., 2008), breast cancer (Parris et al., 2010), cervical cancer (Rajkumar et al., 2011), colorectal cancer (Chen et al., 2010), esophageal cancer (Lin et al., 2006), hepatocellular cancer (Ieta et al., 2007), lung cancer (Kadara et al., 2009), ovarian cancer (Berlingieri et al., 2007), and prostate cancer (Hu et al., 2019). Okamoto et al. found that the overexpression of UBE2C markedly boosts the cell proliferation and migration of NIH 3T3 cells (Okamoto et al., 2003). It has been shown that knockdown of UBE2C reduced the expression of CDK1 and resulted in inhibition of ovarian cancer malignancy (Li et al., 2020). Wang et al. showed that UBE2C has high expression in NSCLC, regulated by miR-548e-5p; UBE2C is able to bind with the 5′UTR of ZEB1/2 and promotes the expression of ZEB1/2, leading to the increase in the cell invasiveness in lung cancer (Jin et al., 2019a). In melanoma, UBE2C was reported to promote the cell growth of melanoma *via* promotion of the G2/M transition (Liu et al., 2019). The above findings suggested that UBE2C plays an important role in the cancer progression and may be a promising prognostic and a therapeutic pan-cancer biomarker. However, the prognosis and immunology role of UBE2C in human cancer have been an uncharted territory.

Epigenetic regulatory mechanisms, including DNA methylation as well as lncRNA, miRNA, and N6-methyladenosine (m6A), were reported to play significant roles in the progression of diverse cancers (Bošković and Rando, 2018; Barbieri and Kouzarides, 2020). For instance, METTL3 facilitates COAD progression by an m6A-IGF2BP2-dependent mechanism, maintaining the stability of SOX2. It has been shown that lncRNA TUC338 activates the MAPK pathway, resulting in promotion of invasion of lung cancer (Zhang et al., 2018a). YTHDF3 promotes the translation of m6A target gene transcripts to promote breast cancer brain metastasis (Chang et al., 2020).

In this study, we utilized the public databases to analyze the expression and prognosis in pan-cancer; our results demonstrate that UBE2C was significantly upregulated in diverse cancers, and its high expression was closely related to the poor prognosis in diverse human cancers. In addition, we found that UBE2C expression was markedly associated with tumor mutational burden (TMB), microsatellite instability (MSI), immune cell infiltration, and diverse drug sensitivities. Finally, we showed that the METTL3/SNHG1/miR-140-3p axis has potential regulation in the expression of UBE2C. The m6A mark improved the stability of methylated SNHG1 transcripts *via* decreasing the RNA degradation rate, which may be a reason for the upregulation of SNHG1 in NSCLC. *In vitro* functional experiments showed that SNHG1, as a competing endogenous RNA, sponges miR-140-3p to increase UBE2C expression in NSCLC cell lines. Our study elucidates the clinical significance and regulatory mechanism of METTL3/SNHG1/miR-140-3p/UBE2C in NSCLC and provides a prognostic indicator as well as a promising therapeutic target for NSCLC patients.

## Materials and Methods

### Analysis of the Expression of UBE2C in Pan-Cancer

We employed the TIMER (https://cistrome.shinyapps.io/timer/) (Li et al., 2017), Oncomine (https://www.oncomine.org), and GEPIA databases (http://gepia.cancer-pku.cn/) (Tang et al., 2017) to analyze the expression of UBE2C in pan-cancer, the Cancer Cell Line Encyclopedia (CCLE) tool (https://portals.broadinstitute.org/ccle/) (Ghandi et al., 2019) was employed to examine the expression of UBE2C in diverse cancer cell lines. The UALCAN tool (http://ualcan.path.uab.edu/) (Chandrashekar et al., 2017) was utilized to analyze the protein of UBE2C in diverse cancers. The expression of miRNA-140-3p was analyzed by Starbase (Li et al., 2014). The Kaplan–Meier plotter (http://kmplot.com/analysis/) (Hou et al., 2017) was employed to examine the prognosis of miRNA-140-3p in pan-cancer.

### Analysis of the Prognosis and Clinical Information of UBE2C in Pan-Cancer

We employed the GEPIA database (http://gepia.cancer-pku.cn/) and PrognoScan database (http://dna00.bio.kyutech.ac.jp/PrognoScan/index.html) (Mizuno et al., 2009; Tang et al., 2017) to analyze the OS and RFS of UBE2C in pan-cancer; additionally, the correlation between the tumor stage and UBE2C expression was analyzed by GEPIA, and the correlation between the tumor stage and UBE2C expression was analyzed by GEPIA. Tumor stage, lymph node metastasis, and expression of miRNA-140-3p were analyzed by the UALCAN tool (http://ualcan.path.uab.edu/) (Chandrashekar et al., 2017). We also employed the prognosis tool to verify the prognosis of UBE2C in pan-cancer.

### Analysis of the Gene Mutation of UBE2C in Pan-Cancer

The gene mutation information of UBE2C in pan-cancer was analyzed by the cBioPortal database (https://www.cbioportal.org/) (Cerami et al., 2012).

### Starbase Database

We employed the Starbase database (http://starbase.sysu.edu.cn/) to forecast the potential miRNAs of UBE2C (Li et al., 2014), and examine the expression, prognosis, and correlation between miRNA-140-3p and UBE2C; we also used Starbase to predict the binding between miRNA, mRNA, and lncRNA.

### Analysis of the Function of UBE2C in Pan-Cancer

We employed the CancerSEA database (http://biocc.hrbmu.edu.cn/CancerSEA/) to analyze the function of UBE2C in pan-cancer (Yuan et al., 2019); LinkedOmics (http://www.linkedomics.org/admin.php) was employed to analyze the KEGG pathway of UBE2C in LUAD (Vasaikar et al., 2018).

### Analysis of the Immunological Roles of UBE2C in Pan-Cancer

We employed the TIMER (https://cistrome.shinyapps.io/timer/) and X-Cell tools (https://xcell.ucsf.edu/) to analyze the immunological roles of UBE2C (Aran et al., 2017; Li et al., 2017), including the correlation between diverse immune cells and immune regulators. TISIDB (http://cis.hku.hk/TISIDB/) was adopted to analyze the relationship between the UBE2C expression and the 28 tumor-infiltrating lymphocytes, 45 immune stimulators, 24 immune inhibitors, 41 chemokines, 18 receptors, and 21 major histocompatibility classes (MHCs) in pan-cancer (Ru et al., 2019). The TMB and MSI scores were obtained from TCGA. A correlation analysis between the UBE2C expression and TMB or MSI was performed using Spearman’s method.

### Analysis of the Correlation Between the UBE2C Expression and Drug Sensitivity

We employed the GDSC and CTRP databases to analyze the correlation between UBE2C expression and drug sensitivity (Basu et al., 2013; Yang et al., 2013).

### Analysis of the Molecular Characteristics of SNHG1

We employed lncLocator (www.csbio.sjtu.edu.cn/bioinf/lncLocator) and CPC2 (http://cpc2.cbi.pku.edu.cn) to examine the subcellular localization and protein-coding ability of SNHG1 (Kang et al., 2017; Cao et al., 2018).

### Plasmid Construction and Cell Culture

The BEAS-2B cell line was purchased from the cell bank of Kunming Institute of Zoology and cultured in BEGM media (Lonza, CC-3170). Lung cancer cell lines, including A549, H1299, and H1975, were purchased from Cobioer, Nanjing, China, with STR document; A549, H1299, and H1975 cells were all cultured in RPMI 1640 medium (Corning, Tewksbury, MA, United States) supplemented with 10% fetal bovine serum (FBS) and 1% penicillin/streptomycin. The siRNAs for METTL3, SNHG1, miRNA mimics NC, and miRNA-140-3p mimics were synthesized by RiboBio, and a scrambled siRNA was synthesized as a negative control. Transfection was performed using Lipofectamine 2000 (Invitrogen, Carlsbad, CA, United States) according to the manufacturer’s instructions. Total RNA was collected 48 h after transfection.

### Quantitative Real-Time PCR

The qRT-PCR assay was performed as documented (Jiang et al., 2018). The primer sequences are listed follows: UBE2C-F: GGA​TTT​CTG​CCT​TCC​CTG​AA, UBE2C-R: GAT​AGC​AGG​GCG​TGA​GGA​AC, METTL3-F: AGC​CTT​CTG​AAC​CAA​CAG​TCC, METTL3-R: CCG​ACC​TCG​AGA​GCG​AAA​T, SNHG1-QPCR-F-GCATCTCATAATCTATCCTGG, SNHG1-QPCR-R-CCTAGTTTTCCTCAAACTCCT, MiRNA-140-3p: TAC​CAC​AGG​GTA​GAA​CCA​CGG, β-actin-F: CTTCGCGGGCGACGAT, β-actin-R: CCA​TAG​GAA​TCC​TTC​TGA​CC. The expression quantification was obtained with the 2−ΔΔCt method.

### Dual-Luciferase Assay

Dual-luciferase assay was performed as documented (Xiong et al., 2021). Briefly, putative binding sites for miR-140-3p on the 3′-UTR of UBE2C were predicted by the Starbase database (http://starbase.sysu.edu.cn/). Wild-type and mutant (mut- UBE2C-3UTR or mut- -3UTR), wild-type, and mutant (mut- SNHG1 or mut- SNHG1) fragments were constructed and inserted downstream of the luciferase reporter gene in the reporter plasmid pGL3 (Promega). A549 and H1975 cells (4×10^4^ cells/well) were seeded in a six-well plate and co-transfected with a 3′-UTR UBE2C construct and miR-140-3p mimics or miR Ctrl using Lipofectamine 2000. Both firefly and Renilla luciferase expressions were measured post-transfection using the Dual-Luciferase Kit (Promega, Madison, WI, United States) according to the manufacturer’s instructions.

### RNA Stability Assays

NSCLC cells were seeded in six-well plates overnight and then treated with actinomycin D (5 μg/ml, HY-17559, MedChemExpress) at 0, 2, 4, and 8 h. Total RNA was then isolated by TRIzol and analyzed by qPCR. The RNA expression for each group at the indicated time was calculated and normalized by β-actin.

### Statistical Analysis

Analysis of the UBE2C expression of pan-cancer was estimated using t-tests. For survival analysis, the HR and p values were calculated utilizing univariate Cox regression analysis. Kaplan-Meier analysis was employed to examine the survival time of patients stratified according to high or low level of the UBE2C expression. For all figures, ∗, ∗∗, and ∗∗∗ indicate *p* < 0.05, *p* < 0.01, and *p* < 0.001, respectively.

## Results

### UBE2C Was Highly Expressed in Human Cancers

**FIGURE 1:**
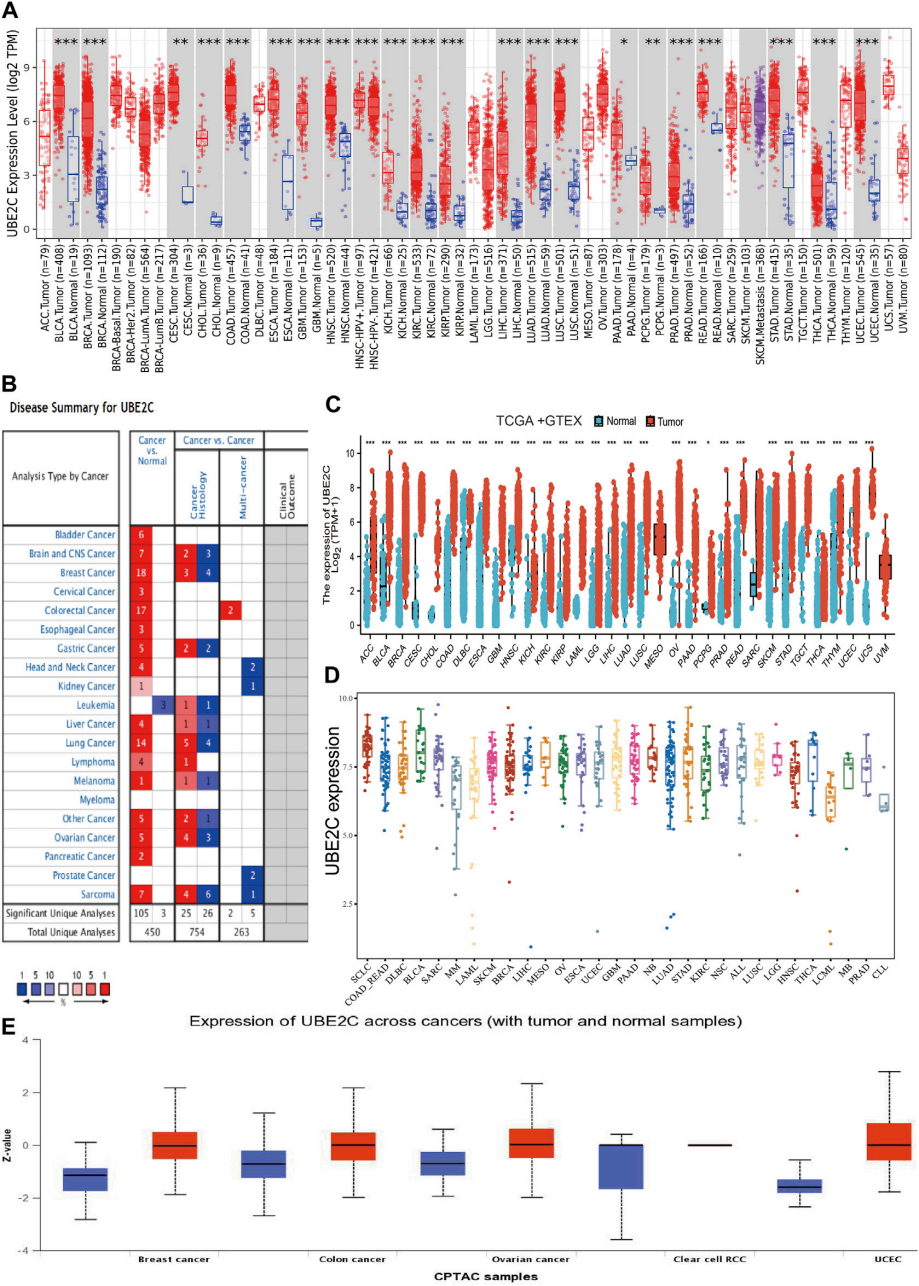
Expression analysis for UBE2C in human cancers. **(A)** The expression of UBE2C in pan-cancer analysis by the TIMER database. **(B)** The expression of UBE2C in pan-cancer analysis by the Oncomine database. **(C)** The expression of UBE2C in pan-cancer examined by the GEPIA database. **(D)** The expression of UBE2C in diverse cancer cell lines examines by the CCLE database. **(E)** The protein expression of UBE2C in pan-cancer analysis by the UALCAN database.

To examine the gene expression of UBE2C, we first used the TIMER database to analyze the levels of UBE2C in various cancers. UBE2C was significantly highly expressed in the following cancers: ACC, BLCA, CESC, CHOL, COAD, ESCA, GBM, HNSC, KICH, KIRC, KIRP, LIHC, LUAD, LUSC, PAAD, PCPG, PRAD, READ, STAD, THCA, and UCEC (Figure 1A). We also examined the expression of UBE2C in human cancer based on GEO data from the Oncomine database. UBE2C was elevated in various cancers, including bladder, brain, CNS, cervical, colorectal, esophageal, gastric, head and neck, liver, lung, ovarian, pancreatic, and prostate cancers, as well as in lymphoma, melanoma, and sarcoma (Figure 1B). Owing to the lack of associated normal tissue data for some cancers in the TCGA databases, we next used the GEPIA database to explore UBE2C expression in human cancers, as shown in Figure 1C; a high expression of UBE2C was observed in the following cancers: ACC, BLCA, BRCA, CESC, COAD, DLBC, ESCA, GBM, HNSC, KICH, KIRC, KIRP, LAML, LGG, LIHC, LUAD, LUSC, OV, PRAD, READ, SKCM, STAD, TGCT, THCA, THYM, UCEC, and UCS. Furthermore, to determine UBE2C expression in diverse cancer cell lines, using the analysis of the CCLE database, we show that UBE2C expression was upregulated in various cancer cell lines (Figure 1D). Finally, in order to verify the above results, we used UALCAN database analysis to investigate the protein levels of UBE2C in human cancer. UBE2C protein was significantly elevated in breast, colon, and ovarian cancers, and in UCEC (Figure 1E). Overall, these results showed that UBE2C was upregulated in diverse human cancers.

### Analysis of the Correlation Between UBE2C Expression and Clinic-Pathologic Features

**FIGURE 2:**
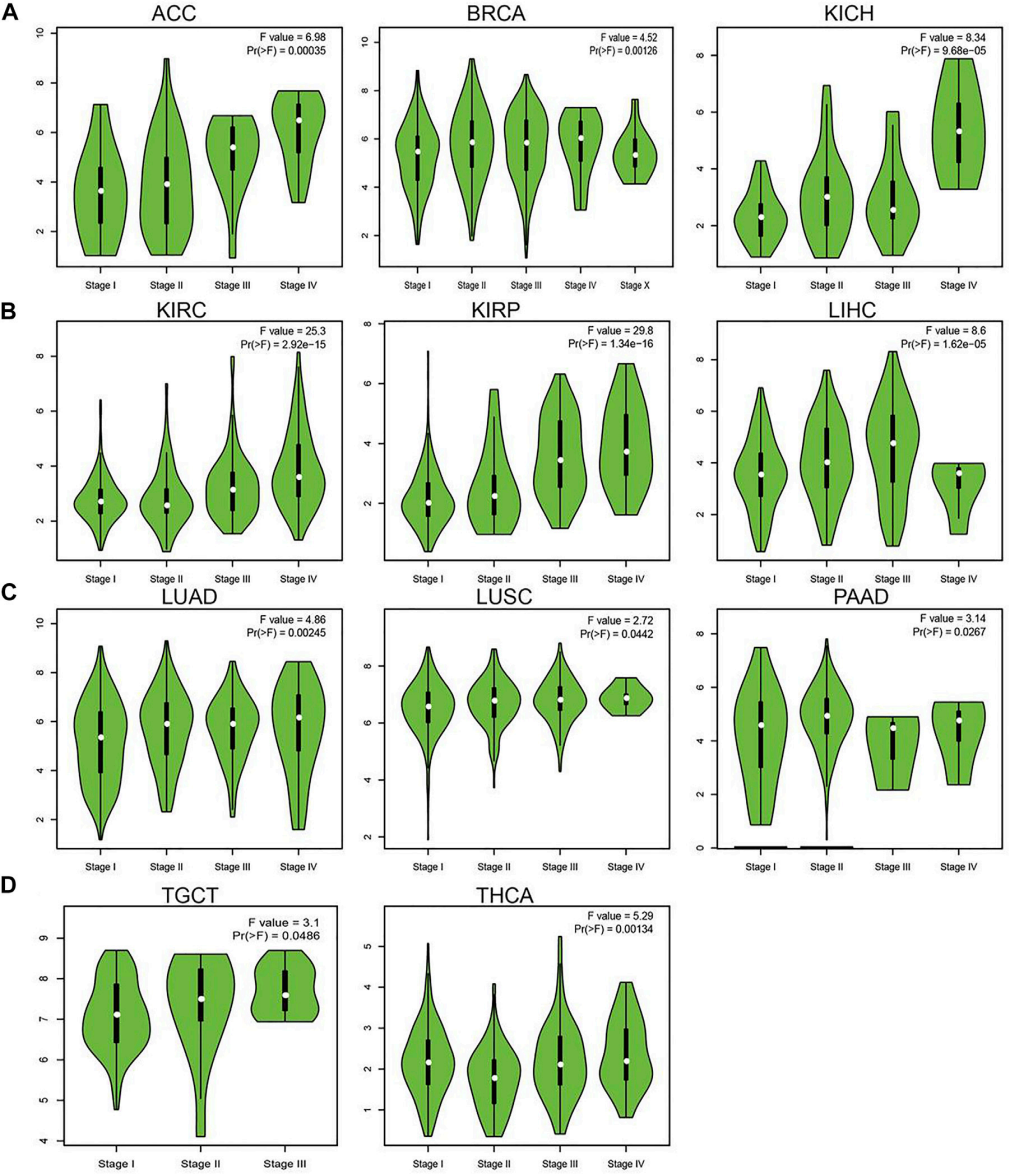
Analysis of the tumor stage for UBE2C in human cancers. **(A)** The tumor stage for UBE2C in ACC, BRCA, and KICH; **(B)** KIRC, KIRP and LIHC; **(C)** LUAD, LUSC, and PAAD; **(D)** and TGCA and THCA analysis by the GEPIA database.

Considering that UBE2C was highly expressed in human cancer, we further explored the correlation between UBE2C expression and the pathological stages of different cancers. The results suggested that the expression of UBE2C was significantly associated with the pathological stage in ACC, BRCA, KICH, KIRC, KIRP, LIHC, LUAD, LUSC, PAAD, TGCT, and THCA (Figures 2A–D). Given that lymph node metastasis plays a crucial role in cancer progression, we next examined the expression of UBE2C in different stages of lymph node metastasis; the results indicated that UBE2C was positively related to the lymph node metastasis of BLCA, BRCA, CESC, CHOL, COAD, ESCA, HNSC, KICH, KIRC, KIRP, LIHC, LUAD, LUSC, MESC, PAAD, PRAD, READ, STAD, and THCA (Supplementary Figures S1A–E). Overall, these findings showed that UBE2C expression was significantly related to clinical pathological characteristics in pan-cancer types.

### Pan-Cancer Analysis of the Prognostic and Diagnostic Value of UBE2C in Pan-Cancer

**FIGURE 3:**
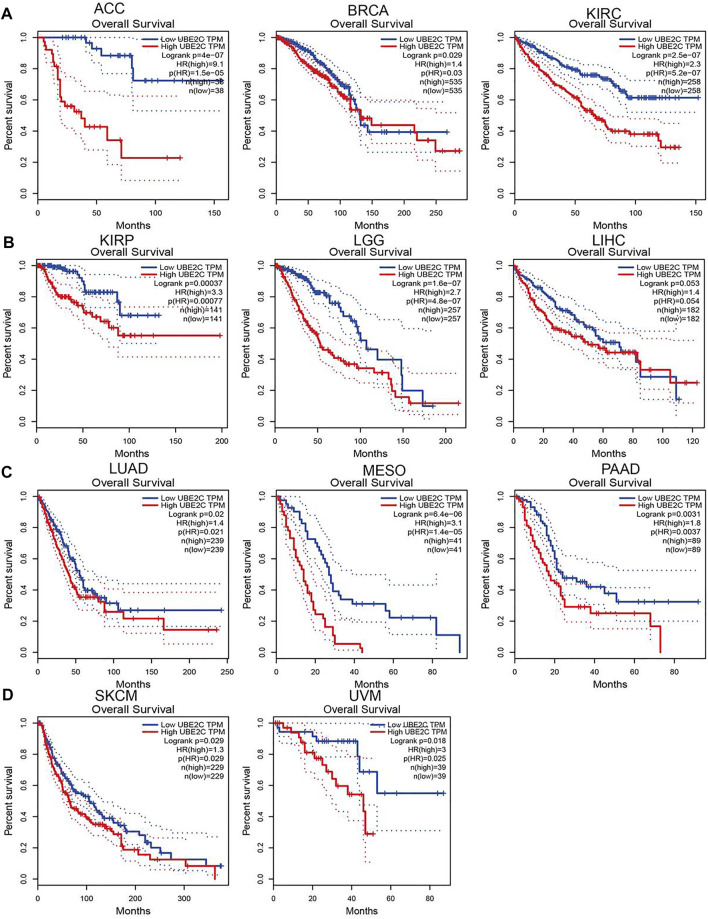
Analysis of the overall survival for UBE2C in human cancers. **(A)** The overall survival for UBE2C in ACC, BRCA and KIRC; **(B)** KIRP, LGG and LIHC; **(C)** LUAD, MESO, and PAAD; **(D)** and SKCM and UVM examined by the GEPIA database.

**FIGURE 4:**
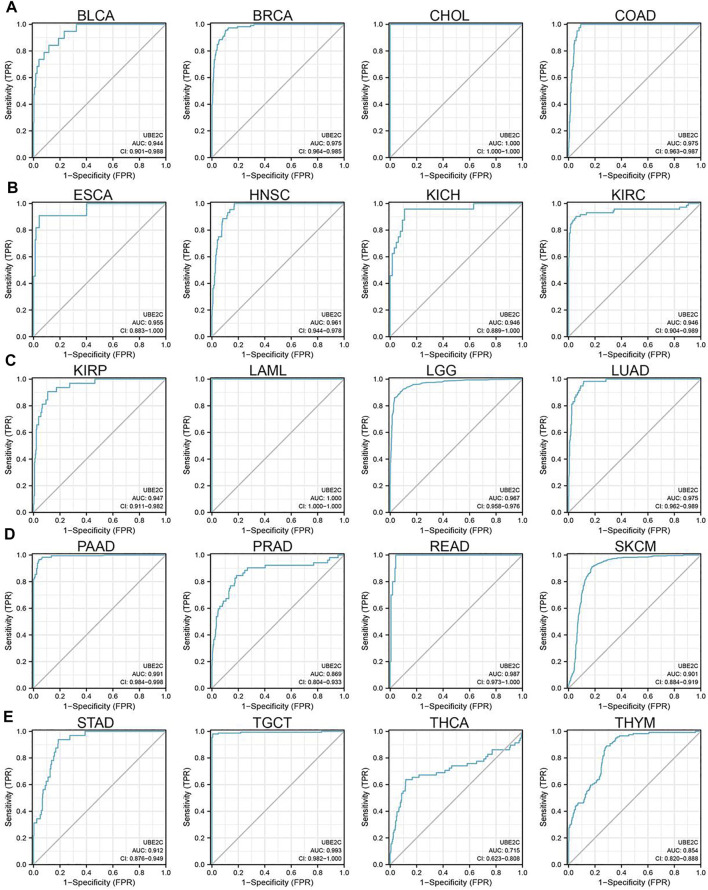
ROC curve analyses and AUC values for UBE2C in diverse cancer. **(A)** ROC curve analysis and AUC values for UBE2C in BLCA, BRCA, CHOL, and COAD. **(B)** ROC curve analysis and AUC values for UBE2C in ESCA, HNSC, KICH, and KIRC. **(C)** ROC curve analysis and AUC values for UBE2C in KIRP, LAML, LGG, and LUAD. **(D)** ROC curve analysis and AUC values for UBE2C in PAAD, PRAD, READ, and SKCM. **(E)** ROC curve analysis and AUC values for UBE2C in STAD, TGCT, THCA, and THYM.

To explore the prognostic role of UBE2C in pan-cancer, we performed OS and RFS analysis in human cancer; the results showed that the high expression of UBE2C was not only related to poor overall survival in ACC, BRCA, KIRC, KIRP, LGG, LIHC, LUAD, MESO, PAAD, SKCM, and UVM (Figures 3A–D and Supplementary Figures S2A–D, S3A–D) but also associated with poor disease-free survival (DFS) in ACC, BRCA, KIRC, KIRP, LGG, LIHC, MESO, PAAD, PRAD, SARC, THCA, and UCEC (Supplementary Figures S2A–D). The Cox regression analysis showed that the high expression of UBE2C was related to poor progression-free survival (PFS) in ACC, KIRC, KIRP, LGG, LIHC, LUAD, MESO, PAAD, PCPG, PRAD, SARC, THCA, UCEC, and UVM. Furthermore, the high expression of UBE2C was associated with poor DSS in CESC, KIRP, LIHC, PRAD, THCA, and UCE2C (Supplementary Figures S2A–D). We previously showed that UCE2C had a high expression and correlated with prognosis in diverse cancer types. Moreover, ROC curve analysis indicated that UCE2C had the potential to act as a biomarker for the diagnosis of diverse cancer types with high sensitivity and specificity (Figures 4A–E).

### Pan-Cancer Analysis of the Correlation Between the UBE2C Expression and Immune, Molecular Subtypes

Human cancer can be divided into various immunological and molecular subtypes; thus, we also examined the UBE2C expression pattern in different cancers. The results showed that UBE2C presents different expression patterns in pan-cancer (Supplementary Figures S4A–D). For example, UBE2C had a high expression in C2 and a low expression in C3. For molecular subtypes, UBE2C displayed a unique expression pattern in cancer (Supplementary Figures S5A–D). These results show that UBE2C has different expression patterns in human cancer.

### Analysis of the Correlation Between UBE2C Expression and Tumor Mutational Burden, and Microsatellite Instability

TMB refers to the number of mutations of DNA in a cancer type and has emerged as a specific and sensitive biomarker of response to immune checkpoint inhibitors (Addeo et al., 2021). We also examined the correlation between UBE2C expression and TMB of human cancer; the results showed that UBE2C expression was markedly positively correlated with the TMB in ACC, PAAD, LUAD, BRCA, PRAD, SARC, LGG, KICH, STAD, BLCA, CHOL, and LUSC and negatively correlated with the TMB in COAD and THYM (Supplementary Figure S6A). MSI represents a hyper-mutable state of DNA sequences caused by the lack of DNA mismatch repair activity (Boland and Goel, 2010). We also explored the correlation between the UBE2C expression and MSI of human cancer, and the results showed that UBE2C expression was markedly positively correlated with the MSI in SARC, MESO, UVM, and ACC and negatively correlated with the MSI in SKCM and THYM (Supplementary Figure S6B).

### Pan-Cancer Analysis of the Functional Role of UBE2C

**FIGURE 5:**
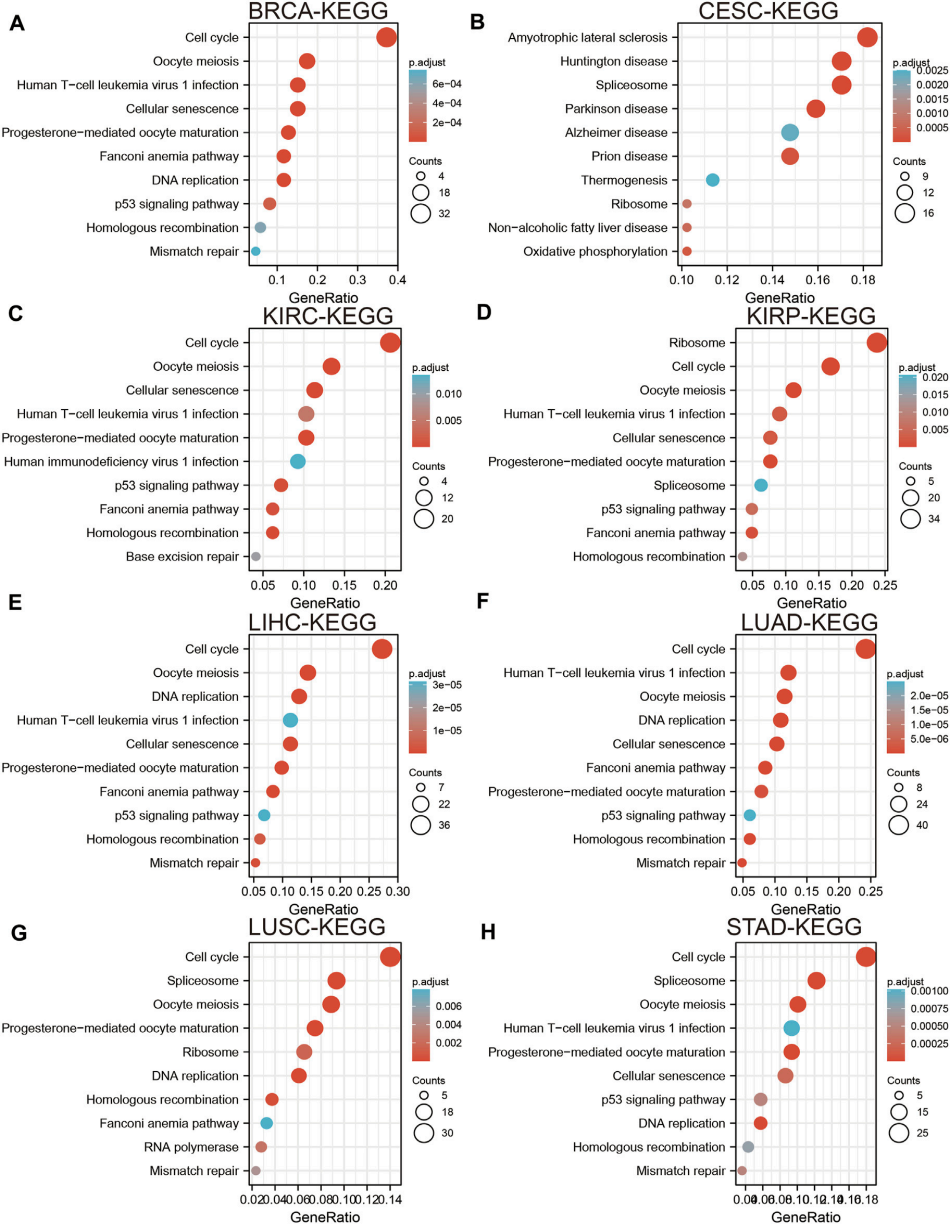
Analysis the signaling pathway for UBE2C in human cancers. **(A–D)** The KEGG pathway of UBE2C in BRCA, CESC, KIRC, and KIRP examine by LinkedOmics. **(E–H)** The KEGG pathway of UBE2C in LIHC, LUAD, LUSC, and STAD examined by LinkedOmics.

To elucidate the biological functions of UBE2C in various cancers, we utilized the LinkedOmics database to perform KEGG-enriched analysis for UBE2C in different cancer types. We found that high expression of UBE2C was mainly associated with the spliceosome, Fanconi anemia pathway, nucleotide excision repair, cellular senescence, and basal transcription factors in BRCA (Figure 5A); oxidative phosphorylation and biosynthesis of amino acids in CESC (Figure 5B); cell cycle checkpoint, DNA recombination, mRNA processing, and RNA modification in KIRC (Figure 5C); the regulation of chromosome organization and ncRNA processing in KIRP (Figure 5D); oocyte meiosis, RNA transport, and cellular senescence in LIHC (Figure 5E); regulation of cell cycle phase transition and DNA metabolism in LUAD (Figure 5F); DNA replication and protein export in LUSC (Figure 5G); and the Hedgehog signaling pathway and histidine metabolism in STAD (Figure 5H). These results indicated that UBE2C plays a crucial role in the development of different cancer types.

### Association of UBE2C With Immune Infiltration in Pan-Cancer

**FIGURE 6:**
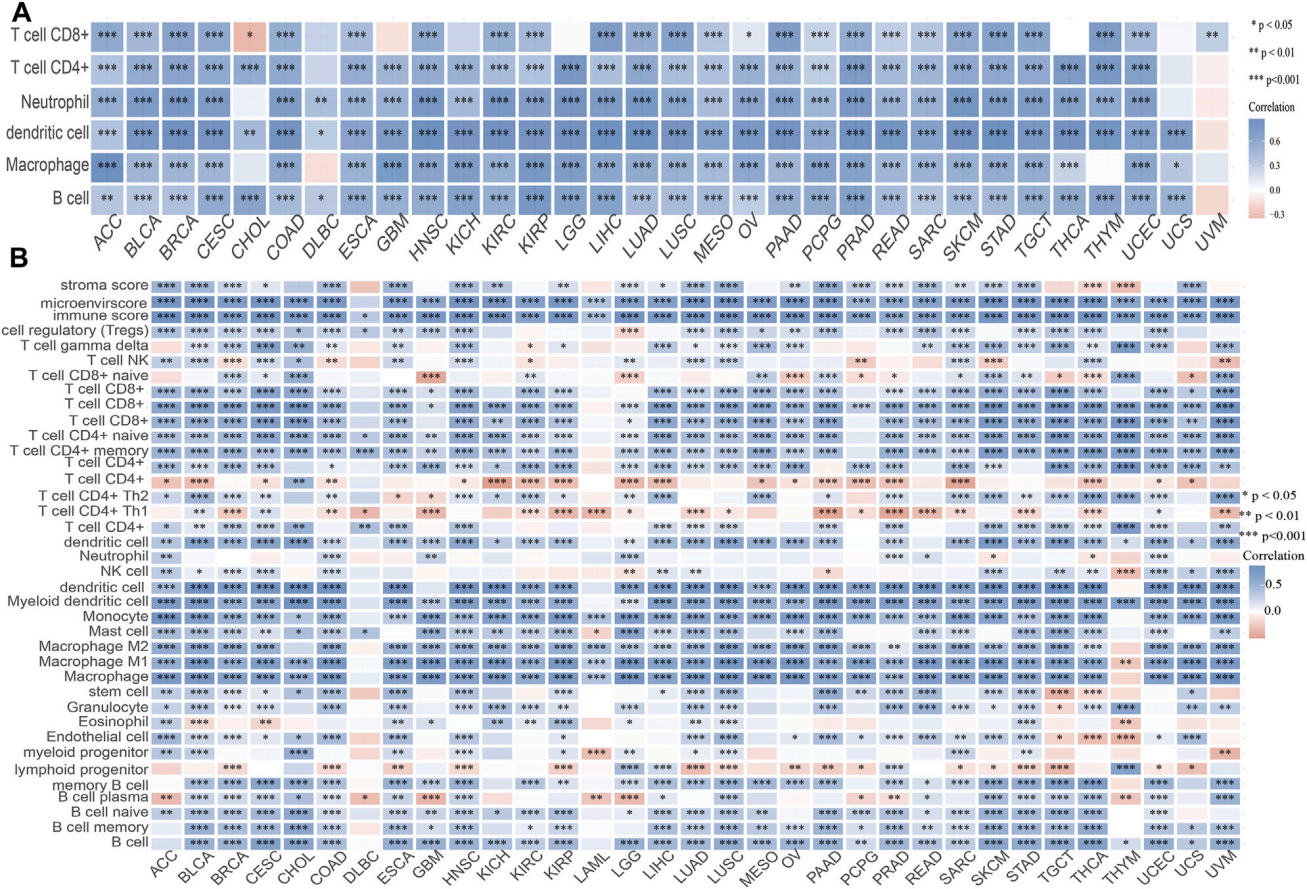
Analysis of the correlation between the UBE2C expression and immune cell infiltration. **(A)** The correlation between UBE2C expression and immune cell infiltration in pan-cancer examined by the TIMER database. **(B)** The correlation between UBE2C expression and diverse immune cells infiltration in pan-cancer examined by X-Cell database. *p < 0.05, **p < 0.01, ***p < 0.001.

Considering that immune cell infiltration plays an indispensable role in cancer progression, we next explored the relationship between UBE2C expression and immune cell infiltration of different cancers. The TIMER analysis results show that UBE2C expression was significantly associated with the abundance of CD8^+^ T cells in 25 cancers, CD4^+^ T cells in 29 cancers, neutrophils in 29 cancers, DCs in 31 cancers, macrophages in 28 cancers, and B cells in 30 cancers (Figure 6A). To further verify the above results, we used the X-Cell tool to examine the correlation between UBE2C expression and immune cell infiltration in various cancers; the results suggested that UBE2C expression was positively correlated with 38 immune cell types in 29 cancers, while it was negatively correlated with 38 immune cell types in four cancers (Figure 6B). These findings indicated that UBE2C was significantly correlated with the infiltration of immune cells in human cancer.

In order to further determine the relationship between UBE2C and the tumor microenvironment (TME), we also examined the correlation between UBE2C and immune checkpoint-related genes by analysis of The Cancer Genome Atlas (TCGA) data. The results suggested that UBE2C expression was positively associated with the immune checkpoint-related genes in 31 cancers, which mainly included CD274, CTLA4, HAVCR2, LAG3, PDCD1, PDCD1LG2, SIGLEC15, and TIGIT (Supplementary Figure S7). The TISIDB tool analysis showed that UBE2C expression was positively associated with the 28 tumor-infiltrating lymphocytes, 45 immune stimulators, 24 immune inhibitors, 41 chemokines, 18 receptors, and 21 MHCs in different cancer types (Supplementary Figures S8A–F). These findings indicated that UBE2C plays an important role in the regulation of the immune response of human cancer.

The TIMER tool analysis shows that UBE2C expression was markedly correlated with the various immune cell gene markers, including B cells (CD19), CD8 + T cells (CD8A, CD8B), DCs (ITGAX, NRP1, CD1C, HLA-DAP1, HLA-DRA, HLA-DQB1, HLA-DPB1), M1 macrophages (PTGS2, IRF5, NOS2), M2 macrophages (MS4A4A, CD163), monocytes (CSF1R, CD86, KIR2DS4, KIR3DL3, KIR3DL2, KIR3DL1), natural killer cells (KIR2DL4,KIR2DL3, KIR2DL1), neutrophils (CCR7, ITGAM, CEACAM8), T cells (CD3D, CD3E, CD2), T cell exhaustion cells (CTLA4, LAG3,HAVCR2, GZMB, PDCD1), TAM cells (CCL2, IL10, CD68), follicular helper (Tfh) cells (BCL6, IL21), Th1 cells (TBX21, STAT4, STAT1, IFNG), Th2 cells (GATA3, STAT6, STAT5A), Th17 cells (STAT3, IL17A), and Treg cells (FOXP3, CCR8, STAT5B, TGFB1). These studies indicated that UBE2C plays a crucial role in the regulation of immune infiltrating cells of pan-cancer (Supplementary Tables S1, S2). We also found that UBE2C expression and different immune cell infiltrations could affect prognosis in different cancer types.

### Correlation Between UBE2C Expression and Diverse Drug Sensitivities

**FIGURE 7:**
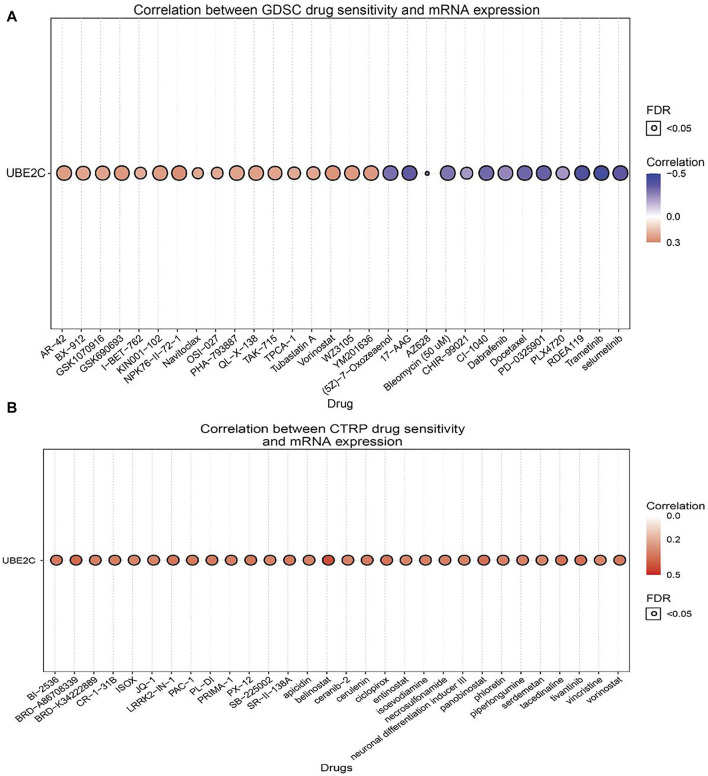
Analysis of the correlation between UBE2C expression and drug sensitivity in diverse human cancer. **(A)** The correlation between UBE2C expression and drug sensitivity in diverse human cancer analysis by the GDSC database. **(B)** The correlation between UBE2C expression and drug sensitivity in diverse human cancer analysis by the CTRP database. *p < 0.05, **p < 0.01, ***p < 0.001.

The above results suggested that UBE2C may act as an oncogene in cancer progression; therefore, we further explored the correlation between UBE2C expression and different drug sensitivities in different cancer cell lines from the GDSC and CTRP databases. The results indicated that the expression of UBE2C was positively correlated with the drug sensitivity of NPK76-II-72-1, vorinostat, WZ3105, YM201636, GSK690693, KIN001-102, AR-42, QL-X-138, GSK1070916, PHA-793887, TAK-715, and BX-912 and was markedly negatively correlated with the drug sensitivity of trametinib, RDEA119, selumetinib, 17-AAG, PD-0325901, docetaxel, CI-1040, AZ628, (5Z)-7-oxozeaenol, bleomycin (50 μM), and bortezomib in the GDSC database (Figure 7A; Supplementary Table S3). In the CTRP database, we observed that UBE2C expression was positively correlated with the drug sensitivity of GSK-J4, belinostat, BRD-A86708339, tivantinib, panobinostat, tacedinaline, ciclopirox, LRRK2-IN-1, PX-12, vorinostat, SR-II-138A, skepinone-L, BI-2536, PAC-1, PL-DI, and cerulenin and negatively associated with the drug sensitivity of BRD-K99006945 dasatinib, trametinib, selumetinib, PD318088, lovastatin, CAY10576, GDC-0879, simvastatin, IC-87114, and fluvastatin (Figure 7B; Supplementary Table S4). In summary, these results suggested that UBE2C was significantly related to drug sensitivity in different cancer cell lines and may be a promising therapeutic target for cancer patients.

### Prediction and Analysis of Upstream miRNAs of UBE2C

**FIGURE 8:**
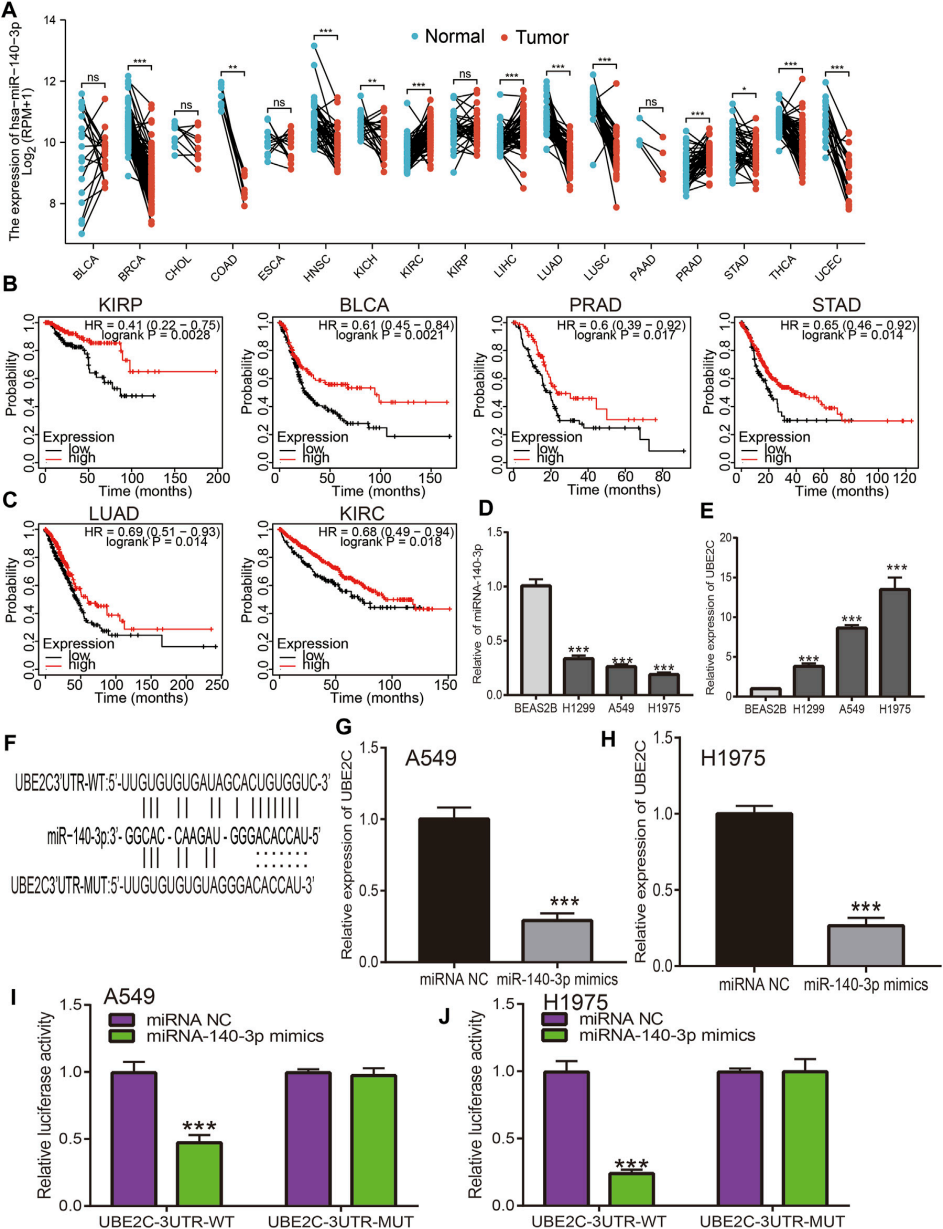
Analysis of the expression and prognosis of miRNA-140-3p in pan-cancer. **(A)** The expression of miRNA-140-3p in pan-cancers. **(B,C)** The prognosis of miRNA-140-3p in pan-cancers examined by kmplot database. **(D,E)** The expression of miRNA-140-3p and UBE2C in NSCLC cell lines examined by qRT-PCR assay. **(F)** The target sites between the UBE2C and miRNA-140-3p predicted by Starbase. **(G,H)** The expression of UBE2C after overexpression of miRNA-140-3p in NSCLC cell lines examined by qRT-PCR assay. **(I,J)** Relative luciferase activities of wild-type (WT) and mutated (MUT) UBE2C-3UTR reporter plasmid in A549 and H1975 cells co-transfected with miR-140-3p mimics examined by luciferase reporter assay. *p < 0.05, **p < 0.01, ***p < 0.001.

**FIGURE 9:**
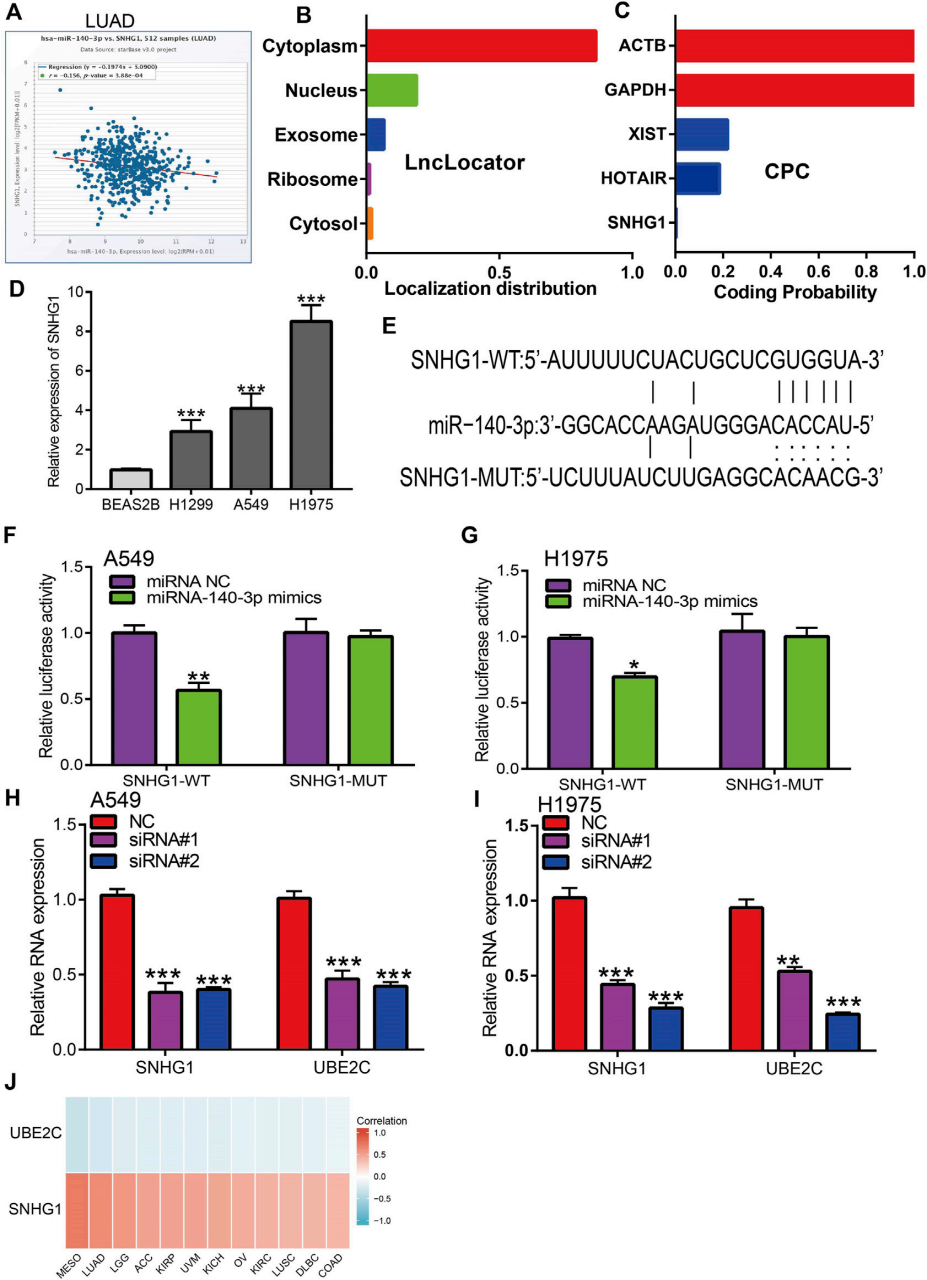
Analysis of upstream lncRNA of miRNA-140-3p in pan-cancer. **(A)** The correlation between the SNHG1 and miRNA-140-3p in LUAD analysis by Starbase. **(B)** The subcellular localization of SNHG1 examined by lncLocator. **(C)** The coding potential of SNHG1 analysis by coding the potential calculator. **(D)** The expression of SNHG1 in NSCLC cell lines examined by qRT-PCR assay. **(E)** The target sites between miRNA-140-3p and SNHG1 was predicted by Starbase. **(F,G)** Relative luciferase activities of wild-type (WT) and mutated (MUT) SNHG1 reporter plasmid in A549 and H1975 cells co-transfected with miR-140-3p mimics examined by luciferase reporter assay. **(H,I)** The expression of UBE2C after depletion of SNHG1 in NSCLC cell lines examined by qRT-PCR assay. **(J)** The correlation between miRNA-140-3p and UBE2C, SNHG1 in NSCLC examined by Starbase. *p < 0.05, **p < 0.01, ***p < 0.001.

MiRNAs play a crucial role in the regulation of mRNA expression; according to the ceRNA theory, miRNA expression should be negatively correlated with mRNA expression, as UBE2C shows high expression in pan-cancer; therefore, regulatory miRNA expression should be lower in cancer. We next utilized diverse public databases to predict the upstream miRNAs of UBE2C. We obtained four miRNAs (hsa-miR-17-5p, hsa-miR-889-3p, hsa-miR-513b-5p, and miR-140-3p) that intersected with UBE2C expression. Among these miRNAs, only miR-140-3p was significantly decreased in human cancers (Figure 8A). Next, we analyzed the expression and prognosis of miR-140-3p in pan-cancer; the results showed that miR-140-3p had a low expression in various cancers. In contrast, a high expression of miR-140-3p was observed in KIRC and LIHC. Furthermore, a lower expression of miR-140-3p was correlated with poor prognosis in KIRP, BLCA, PRAD, STAD, LUAD, KIRC, READ, THYM, and CESC (Figures 8B,C). Further studies showed that miR-140-3p expression was significantly associated with the occurrence of lymph node metastasis in different cancers (Supplementary Figures S9A–E). The ROC curve analysis showed that miR-140-3p had the potential to act as a prognostic marker in the diagnosis of different human cancers (Supplementary Figures S10A–C). We also performed a pan-cancer examination of the correlation between miR-140-3p and UBE2C; the results showed that miR-140-3p was negatively correlated with the expression of UBE2C in human cancer (Figure 9J). To verify the above hypothesis, we used NSCLC cell lines. We found that UBE2C was highly expressed and miR-140-3p downregulated in NSCLC cell lines (Figures 8D,E). We also found that UBE2C could be influenced by the expression of a miR-140-3p mimic, whereby the overexpression of miR-140-3p could significantly reduce the expression of UBE2C in NSCLC cells, including A549 and H1975 cells (Figures 8F–H). Additionally, luciferase reporter assays showed that the overexpression of miR-140-3p significantly repressed luciferase activity in NSCLC cells transfected with the wild-type (UBE2C-3UTR WT) reporter plasmid, whereas no obvious inhibition was observed in cells transfected with the mutant reporter plasmid (UBE2C-3UTR MUT) (Figures 8I,J). Consequently, these results suggested that miR-140-3p could bind to the UBE2C 3′UTR promoter region in NSCLC.

### Prediction and Analysis of miR-140-3p Upstream lncRNAs

We performed a pan-cancer analysis to determine the upstream lncRNAs of miR-140-3p. We employed the Starbase and lncRNABase tools to predict potential lncRNAs that bind to miR-140-3p. We identified four possible lncRNAs, including TUG1, SNHG1, and MIR663AHG. Of these lncRNAs, only SNHG1 was significantly upregulated in various human cancers (Supplementary Figure S11A) and a high expression of SNHG1 was associated with a good prognosis in THYM, STAD, READ, OV, LUAD, HNSC, ESCA, BLCA, and LGG, but was associated with a poor prognosis in UCEC, SRAC, LIHC, KIRP, KIRC, ACC, and STAD, while the expression of other lncRNA showed no significant differences in other cancer types (Supplementary Figures S11B–E). ROC curve analysis showed that SNHG1 had the potential to act as a biomarker for the diagnosis of ACC, BLCA, CHOL, COAD, ESCA, GBM, HNSC, KIRC, KIRP, LAML, LGG, NSCLC, OV, PRAD, READ, and SKCM (Supplementary Figures S12A–D).

Based on the ceRNA theory, lncRNA should be negatively correlated with miR-140-3p and positively correlated with UBE2C expression, a condition that was fully satisfied by the expression of SNHG1. We found that SNHG1 exhibited a significant negative correlation with the expression of miR-140-3p (Figures 9A,J) and a positive correlation with the expression of UBE2C in the pan-cancer analysis. We performed the subcellular localization of SNHG1 and potential coding analysis using different public databases. The subcellular localization analysis using the lncLocator tool indicated that SNHG1 was mainly located in the cytoplasm of cells (Figure 9B). We also analyzed the coding potential of SNHG1 using a coding potential calculator, and the results showed that SNHG1 did not possess the protein-coding ability (Figure 9C).

To verify the above hypothesis, we used NSCLC cell lines to evaluate SNHG1 expression. SNHG1 was highly expressed in NSCLC cell lines (Figure 9D). Furthermore, target-binding sites of miR-140-3p on SNHG1 were predicted using Starbase (Figure 9E). In addition, luciferase reporter assays showed that the overexpression of miR-140-3p significantly repressed luciferase activity in NSCLC cells transfected with the wild-type (SNHG1-WT) reporter plasmid, whereas no obvious inhibition was observed in cells transfected with the mutant reporter plasmid (SNHG1-MUT) (Figures 9F,G). Finally, we found that UBE2C could be influenced by changes in SNHG1 expression: SNHG1 knockdown significantly reduced UBE2C expression in NSCLC cells, including A549 and H1975 cells (Figures 9H,I). Overall, these results partially demonstrate that SNHG1 may act as a potential sponge for miR-140-3p and modulates the expression of UBE2C in NSCLC cells.

### M6A Modifications Improved the Stability of the SNHG1 RNA *via* an m6A-Dependent Manner in NSCLC

**FIGURE 10:**
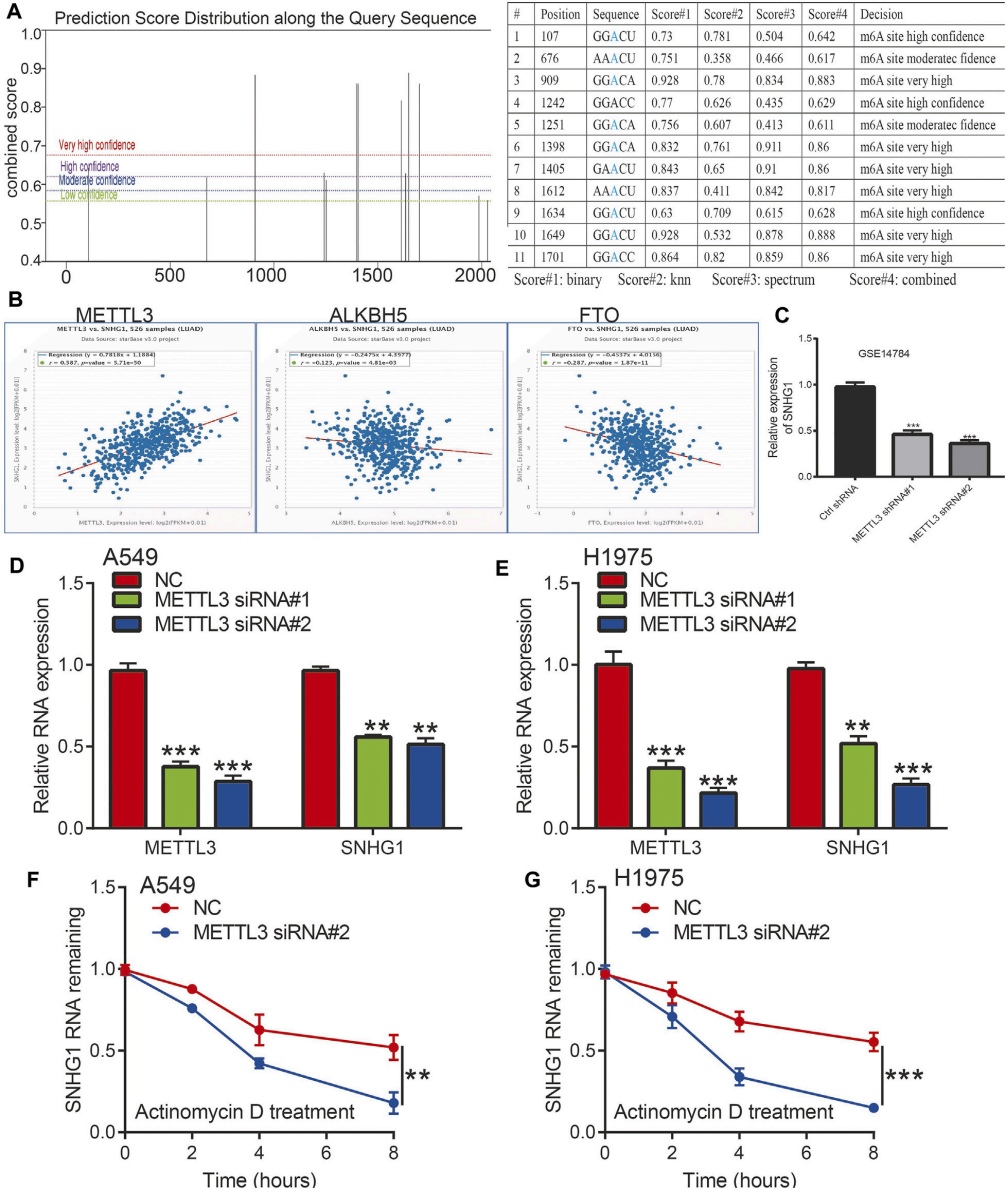
The m6A modification is enriched in SNHG1 and improves its transcript stability. **(A)** The RNA m6A methylation site distribution. **(B)** The correlation between SNHG1 and METTL3, ALKBH5, and FTO in TCGA LUAD. **(C)** The expression of SNHG1 after depletion of METTL3 analysis by the GEO database. **(D,E)** The expression of SNHG1 after depletion of METTL3 in NSCLC cell lines examined by qRT-PCR assay. **(F,G)** METTL3 knockdown in A549 and H1975 cells significantly downregulated SNHG1 RNA abundance. *p < 0.05, **p < 0.01, ***p < 0.001.

Recently, emerging evidence has demonstrated that m6A modification in ncRNAs is extremely widespread and functionally modulates the eukaryotic transcriptome to influence RNA splicing, export, localization, and stability (Zhang et al., 2017; Coker et al., 2019; Wang et al., 2019; Wu et al., 2019a). To study whether m6A modifications were present on SNHG1, we first used the SRAMP database to predict potential m6A methylation sites in the transcript of SNHG1, and several m6A methylation sites in the transcript of SNHG1 were detected (Figure 10A). To determine the relationship between the m6A methylation of RNA and SNHG1 expression, we analyzed the correlation between the levels of m6A methyltransferase and m6A demethylases; the results demonstrated that SNHG1 was significantly positively or negatively correlated with the expression of METTL3, ALKBH5, and FTO, respectively (Figure 10B). A previous study also demonstrated that METTL3 could maintain the stability of lncRNA and promoted NSCLC drug resistance and metastasis (Jin et al., 2019b). We analyzed the GEO data set and found that METTL3 knockdown significantly reduced the SNHG1 expression levels (Figure 10C).

We hypothesized that m6A modification allows maintenance of SNHG1 RNA stability and was upregulated in NSCLC. To verify the above hypothesis, we used the NSCLC cell lines to perform siRNA-mediated silencing of METTL3, a core component of the m6A methylase complex (Zeng et al., 2020). Depletion of METTL3 significantly reduced the expression of SNHG1 in NSCLC (Figures 10D,E). We then examined the loss of SNHG1 RNA after blocking new RNA synthesis with actinomycin D. The results showed that SNHG1 presented reduced RNA stability after knockdown of METTL3 (Figures 10F,G). These findings raise the possibility that the modification of m6A in SNHG1 could improve the stability of the transcripts, which may partially explain the significantly increased regulation of SNHG1 in NSCLC.

## Discussion

Emerging evidence has shown that UBE2C plays an indispensable role in ubiquitin conjugation, regulation of cell cycle progression, mitotic spindle checkpoints, and cancer progression. UBE2C is necessary for the correlated activity between mitotic cyclins and its substrates (Aristarkhov et al., 1996). During the cell metaphase, UBE2C promotes cell cycle progression by interfering with cyclin B (Rape et al., 2006). Our pan-cancer analysis indicated that UBE2C mRNA was decreased in the majority of normal tissues, while UBE2C was significantly elevated in cancer tissues, such as in colon cancer and lung cancer (Hao et al., 2012). The high expression of UBE2C was associated with a poor prognosis in breast, colon, liver, lung, and ovarian cancer (van Ree et al., 2010). The molecular mechanism regulating the upregulation of UBE2C in different cancers is largely unresolved (Nath et al., 2011). One study showed that gene amplification may be a potential mechanism (Vasiljevic et al., 2013). Another possible mechanism is upregulation by key transcription factors, such as MED1 and c-Myc, in prostate cancer and breast cancer, respectively (Hao et al., 2012).

UBE2C has been described as a molecular marker for human lung cancer and liver cancer prognosis (Kadara et al., 2009; Wu et al., 2019b), while another study indicated that UBE2C could be a potential biomarker for tumorigenesis and prognosis in squamous cell carcinoma of the tongue (Liu et al., 2020a). Wahafu et al. reported that higher expression of UBE2C predicts severe outcomes and resistance to therapy in patients with glioma (Alafate et al., 2019). Yong et al. reported that UBE2C may be a clinically independent prognostic factor for patients with ccRCC (Luo et al., 2019). Hao et al. found that the expression of UBE2C in gastric cancer tissues is significantly greater than in adjacent normal tissues. UBE2C expression has been correlated with lymphatic metastasis, serosa invasion, TNM staging, and Lauren’s classification. The univariate and multivariate analyses demonstrate that UBE2C expression, lymphatic metastasis, and TNM staging are independent prognostic indicators in gastric cancer (Zhang et al., 2018b). In our study, using various public databases, such as GEO, CCLE, GEPIA, UALCAN, TIMER, and Oncomine, we found that UBE2C was upregulated in different human cancers, and high expression of UBE2C was significantly associated with tumor stage and lymph node metastasis of various cancers. In addition, high expression of UBE2C was associated with poor prognosis of human cancer. The ROC curve analysis indicated that UCE2C had the potential to act as a biomarker for the diagnosis of diverse cancer types with high sensitivity and specificity. Therefore, these findings provide strong evidence that UCE2C is an attractive target for cancer therapy and may lead to the discovery of UCE2C inhibitors to treat cancer and prevent drug resistance.

TMB and MSI emerged as a specific and sensitive biomarker of response to immune checkpoint inhibitors (Jin et al., 2019a).Zhang et al. (2018b) reported that the chromosomal instability-related gene UBE2C was a potential biomarker of intestinal-type gastric cancer. The overexpression of the UBE2C protein predicted a poor clinical outcome. In this study, we found that UBE2C was significantly associated with the TMB and MSI in different cancers. With regard to the function of UBE2C in cancer, recent studies have shown that UBE2C was overexpressed in NSCLC tissues compared to normal tissues and high expression of UBE2C was correlated with advanced pathological stage, while UBE2C knockdown significantly inhibited cell growth and promoted NSCLC cell apoptosis (Zhang et al., 2015). Furthermore, studies showed that UBE2C was upregulated in endometrial cancer and associated with advanced histologic grade, FIGO stage, recurrence, and shorter overall survival. Depletion of UBE2C inhibits endometrial cancer cell proliferation, migration, invasion, and epithelial–mesenchymal transformation (EMT), whereas UBE2C overexpression exerted opposite effects (Liu et al., 2020b). Further study showed that estradiol induced UBE2C expression *via* estrogen receptor α, which binds directly to the UBE2C promoter element. Conversely, high expression of UBE2C enhanced p53 ubiquitination to facilitate its degradation in endometrial cancer cells (Liu et al., 2020b). In addition, UBE2C has been found to be upregulated in liver cancer, and depletion of UBE2C obviously suppressed the proliferation, migration, and invasion of HCC and improved the sensitivity of HCC cells to sorafenib (Xiong et al., 2019). Sheu et al. found that UBE2C was overexpressed in human CESC tissues and its expression was associated with the clinical characteristics of CESC patients. The depletion of UBE2C reduced cervical cancer cell proliferation by inhibiting the mTOR/PI3K/AKT pathway (Chiang et al., 2020). Hu et al. reported that UBE2C was overexpressed in HNSCC cells and inhibition of UBE2C expression significantly suppressed the malignant phenotypes of HNSCC cells *in vitro* (Jin et al., 2020). In this study, our KEGG analysis showed that the high expression of UBE2C mainly involved the regulation of chromosome organization, cell cycle phase transition, and the Hedgehog signaling pathway. These results indicated that UBE2C plays a crucial role in the initiation and progression of different cancer types.

In recent years, immunotherapy to boost T cell functionality in tumors has rapidly become a standard treatment. Kastenmüller et al. reported that CD4^+^ T cells play crucial roles in cancer immunology and immunotherapy (Borst et al., 2018). CD4^+^ and CD8^+^ T cells, as a part of the cancer immune cycle, both significantly influence the clinical outcome (Ostroumov et al., 2018); CD4^+^ T cells secrete a variety of cytokines that have direct effector functions and activate other immune cells (Swain et al., 2012). A further example is that, in lung cancer, tumor-infiltrating CD4^+^ T cells play an indispensable role in the immune response. CD4^+^ T cells kill cancer cells by allowing CD8 + T cell entry to tumor sites or mucosa (Bos and Sherman, 2010). Considering the importance of the TME in the progression of cancer, our results show that UBE2C expression was markedly positively correlated with the diverse immune cell infiltration in human cancer. In addition, UBE2C expression was significantly upregulated in 28 tumor-infiltrating lymphocytes, 45 immune stimulators, 24 immune inhibitors, 41 chemokines, 18 receptors, and 21 MHCs in pan-cancer. These findings indicated that UBE2C plays an indispensable role in the regulation of the immune response of human cancers.

Preclinical and clinical studies have shown that inhibitory immune checkpoint molecules, including CD274, CTLA4, HAVCR2, LAG3, PDCD1, PDCD1LG2, SIGLEC15, and TIGIT, are elevated during the course of sepsis. Programmed death-ligand 1 (PD-L1) is frequently observed in different cancers. PD-1 could directly bind with PD-L1 and activate T cells, which inhibited antitumor immunity by counteracting T cell-activating signals (Sun et al., 2018). Indeed, studies have shown that therapeutic agents aimed at blocking the engagement of inhibitory immune checkpoints on immune cells play a crucial role in improving innate and adaptive immune cell functions, increasing host resistance to infection and significantly improving survival (Patil et al., 2017). In this study, we found that UBE2C expression was positively associated with an immune checkpoint-related gene in 31 cancer types. These immune checkpoint-related genes included CD274, CTLA4, HAVCR2, LAG3, PDCD1, PDCD1LG2, SIGLEC15, and TIGIT. Therefore, overall, our findings showed that UBE2C might influence immune cell infiltration, which makes it a predictive biomarker for immunotherapy in cancer patients.

UBE2C expression was also positively correlated with drug sensitivity of NPK76-II-72-1, vorinostat, WZ3105, YM201636, GSK690693, KIN001-102, AR-42, QL-X-138, GSK1070916, PHA-793887, TAK-715, and BX-912 and was markedly negatively correlated with the drug sensitivity of trametinib, RDEA119, selumetinib, 17-AAG, PD-0325901, docetaxel, CI-1040, AZ628, (5Z)-7-oxozeaenol, bleomycin (50 μM), and bortezomib in the GDSC database.

NcRNAs, such as miRNAs and lncRNAs, are involved in the regulation of gene expression *via* ceRNA regulatory mechanisms. For example, Wang et al. found that lncRNA SNHG1-sponged miR-154-5p and higher EZH2 expression led to the modulation of colorectal cancer cell growth (Xu et al., 2018). Song et al. reported that SNHG1 was highly expressed in cervical cancer tissues and cervical cancer cell lines, while depletion of SNHG1 significantly inhibited cell proliferation and migration as well as invasiveness of HeLa and C-33A cell lines (Liu et al., 2018). Xu et al. have shown that SNHG1 contributes to cell proliferation and invasion by inhibiting miR-382 expression in breast cancer (Zheng et al., 2019). In cervical cancer, UBE2C was reported to be a target gene of miR-525-5p, and overexpression of miR-525-5p dramatically repressed cell viability, invasiveness, and migration ability; elevated the expression of UBE2C; and partially blunted the salutary effects of miR-525-5p on invasive ability (Chen and Liu, 2020).

To explore the upstream regulatory miRNAs of UBE2C, we obtained four miRNAs, including hsa-miR-17-5p, hsa-miR-889-3p, hsa-miR-513b-5p, and miR-140-3p. These miRNAs play different roles in cancer progression. For example, miR-17-5p expression has been shown to decrease in primary CRC tissues; overexpression of miR-17-5p significantly represses cell migration and invasion by modulating vimentin expression in colorectal cancer (Kim et al., 2020). Fu et al. found that miR-17-5p modulates PTEN expression and as a result promotes the drug resistance and invasion of ovarian carcinoma cells (Fang et al., 2015). Zhang et al. found that a high level of miR-17-5p promotes migration and invasion of cervical cancer cells by regulating the TIMP2/MMP signaling cascade (Zou and Zhang, 2021). Wang et al. found that miR-17-5p was highly expressed in lung cancer, and its high expression was correlated with poor prognosis (Chen et al., 2013). With regard to miR-889-3p function, lncRNA XIST modulates the miR-889-3p/SIX1 axis and participates in cervical cancer progression (Liu et al., 2020c). Yu et al. found that hsa_circ_0009035 inhibits cervical cancer progression and enhances radiosensitivity by interacting with miR-889-3p and suppressing HOXB7 (Zhao et al., 2021). Furthermore, circ_LARP4 suppresses cell proliferation and migration in ovarian cancer by upregulation of LARP4 by competitive binding to miR-513b-5p (Lin et al., 2020). Sun et al. reported that miR-513b-5p overexpression suppresses cell proliferation through upregulation of IRF2 in testicular embryonal carcinoma cells (Wang et al., 2017). In hepatocellular carcinoma, miR-513b-5p inhibits PIK3R3 expression and represses autophagy (Jin et al., 2021). Huang et al. found that miR-140-3p inhibits the progression of osteoarthritis by regulating the expression of CXCR4 (Ren et al., 2020). Koike et al. reported that miR-140 regulated the expression of NF-κB coactivators, which resulted in the suppression of NF-κB activity (Takata et al., 2011). After performing correlation analysis, expression analysis, survival analysis, and luciferase activity assays, we can confirm that miR-140-3p was the most likely upstream tumor suppressor miRNA of UBE2C. Previous studies have also shown that miR-140-3p had a lower expression in NSCLC, and its overexpression reduced the migration and invasion properties of NSCLC cells (Flamini et al., 2017). In this study, we found that miR-140-3p was negatively correlated with UBE2C in a pan-cancer analysis. MiR-140-3p was decreased in different cancers and was correlated with poor prognosis. Moreover, miR-140-3p had a low expression in NSCLC cell lines and could modulate UBE2C expression.

Based on the ceRNA hypothesis, potential lncRNAs of the miR-140-3p/UBE2C axis may act as oncogenic lncRNAs in multiple cancer types. We also identified four upstream lncRNAs of the miR-140-3p/UBE2C axis: TUG1, SNHG1, SCARNA2, and MIR663AHG.

SNHG1 has been reported to function as oncogene in multiple malignancies, including NSCLC. For example, lncRNA SNHG1 has been shown to modulate the miR-361-3p/FRAT1 axis and to promote cell proliferation, migration, invasion, and apoptosis of NSCLC cells (Li and Zheng, 2020). Gao et al. found that the lncRNA SNHG11 by activation of the Wnt/β-catenin signaling pathway promotes the proliferation and migration of lung cancer cells (Liu et al., 2020d). Additionally, Tao et al. showed that long noncoding RNA SNHG1 promoted the progression of NSCLC (Lu et al., 2018).

Emerging evidence has demonstrated that TUG1 plays a critical role in cancer progression. For example, Xu et al. found that lncRNA TUG1 *via* interaction with miR-145 promotes papillary thyroid cancer cell proliferation, migration, and EMT formation (Lei et al., 2017). Liu et al. found that lncRNA TUG1 *via* upregulation of AURKA accelerates cell proliferation and inhibits apoptosis (Li et al., 2018). Zhao et al. found that lncRNA TUG1 promotes the progression of prostate cancer and predicts poor prognosis (Xu et al., 2019). Gou et al. found that lncRNA TUG1 promotes prostate cancer cell proliferation, invasion, and migration by regulating the Nrf2 signaling axis (Yang et al., 2020). Shao et al. found that TUG1 downregulates KLF4 expression and facilitates the metastasis and the EMT of colorectal cancer by miR-153-1 (Shao et al., 2019). Furthermore, Zhang et al. showed that SCARNA2 mediates colorectal cancer chemoresistance through interaction with mirR-342-3p (Zhang et al., 2019). Ma et al. showed that SCARNA2 induces cutaneous squamous cell carcinoma progression by regulating miR-342-3p expression (Zhang et al., 2020). Previous studies have shown that METTL3 methylated SOX2 transcripts, subsequently recognized by the specific m6A “reader” and insulin-like growth factor 2 mRNA-binding protein 2 (IGF2BP2), to maintain SOX2 stability and promote colorectal carcinoma progression (Li et al., 2019). METTL3-mediated modification of m6A has been shown to induce the upregulation of LINC00958 by stabilizing its RNA transcripts and promoting HCC lipogenesis and progression (Zuo et al., 2020). The above results confirm that m6A modification plays a crucial role in the stability of the RNAs. In this study, by performing expression analysis, survival analysis, correlation analysis, and luciferase activity assays, a potential upregulated lncRNA, SNHG1, was identified. We found that SNHG1 was overexpressed in our pan-cancer analysis and correlated with poor prognosis, including that of NSCLC. To elucidate the abnormal high expression mechanism of SNHG1 in NSCLC, we further found that the modification of m6A was enriched in the SNHG1 RNA, and the modification of m6A in SNHG1 leads to an improvement in its RNA stability, which may partially explain the upregulation of SNHG1 in NSCLC. Taken together, the SNHG1/miRNA-140-3p/UBE2C axis was identified as a potential regulatory pathway in NSCLC.

## Conclusion

**FIGURE 11:**
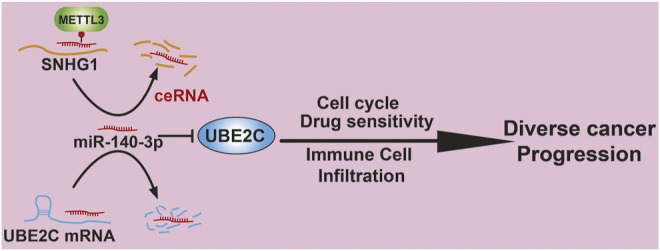
Proposed model of the mechanism underlying the expression and function of METTL3/lncRNA-SNHG1/miRNA-140-3p/UBE2C Axis in Pan-cancer.

In summary, our study showed that UBE2C was elevated in multiple types of human cancer and positively correlated with an unfavorable prognosis. In addition, a high expression of UBE2C was associated with TMB, MSI, immune cell infiltration, and diverse drug sensitivities. Finally, we identified an upstream regulatory mechanism of UBE2C in NSCLC, namely, the METTL3/SNHG1/miR-140-3p axis (Figure 11). Herein, we provided the first evidence to show that the METTL3/SNHG1/miR-140-3p/UBE2C axis plays a crucial role in cancer progression and the immune response in human pan-cancer.

## Data Availability

The datasets presented in this study can be found in online repositories. The names of the repository/repositories and accession number(s) can be found in the article/Supplementary Material.

## Glossary

Cancer Type

ACC: adrenocortical carcinoma
BLCA: bladder urothelial carcinoma
BRCA: breast invasive carcinoma
CESC: cervical squamous cell carcinoma and endocervical adenocarcinoma
CHOL: cholangiocarcinoma
COAD: colon adenocarcinoma
DLBC: lymphoid neoplasm diffuse large B-cell lymphoma
ESCA: esophageal carcinoma
GBM: glioblastoma multiforme
HNSC: head and neck squamous cell carcinoma
KICH: kidney chromophobe
KIRC: kidney renal clear cell carcinoma
KIRP: kidney renal papillary cell carcinoma
LAML: acute myeloid leukemia
LGG: brain lower grade glioma
LIHC: liver hepatocellular carcinoma
LUAD: lung adenocarcinoma
LUSC: lung squamous cell carcinoma
MESO: mesothelioma
OV: ovarian serous cystadenocarcinoma
PAAD: pancreatic adenocarcinoma
PCPG: pheochromocytoma and paraganglioma
PRAD: prostate adenocarcinoma
READ: rectum adenocarcinoma
SARC: sarcoma
SKCM: skin cutaneous melanoma
STAD: stomach adenocarcinoma
TGCT: testicular germ cell tumors
THCA: thyroid carcinoma
THYM: thymoma
UCEC: uterine corpus endometrial carcinoma
UCS: uterine carcinosarcoma
UVM: uveal melanoma
