# Corrigendum: Comprehensive pan-cancer analysis of the prognostic and immunological roles of METTL3/lncRNA-SNHG1/miRNA-140-3p/UBE2C axis

**DOI:** 10.3389/fcell.2022.1009752

**Published:** 2022-08-25

**Authors:** Xiulin Jiang, Yixiao Yuan, Lin Tang, Juan Wang, Qianqian Liu, Xiaolan Zou, Lincan Duan

**Affiliations:** ^1^ Key Laboratory of Animal Models and Human Disease Mechanisms of Chinese Academy of Sciences & Yunnan Province, Kunming Institute of Zoology, Kunming, China; ^2^ Department of Thoracic Surgery, The Third Affiliated Hospital of Kunming Medical University, Kunming, China

**Keywords:** m6A modification, SNHG1, miRNA-140-3p, UBE2C, pan-cancer, prognosis, immune cell infiltration, drug sensitivity

In the original article there was a mistake in the **author affiliations** for Xiulin Jiang which was erroneously presented as “Department of Thoracic Surgery, The Third Affiliated Hospital of Kunming Medical University, Kunming, China”, it should instead be “Key Laboratory of Animal Models and Human Disease Mechanisms of Chinese Academy of Sciences & Yunnan Province, Kunming Institute of Zoology, Kunming, China”.

The authors apologize for this error and state that this does not change the scientific conclusions of the article in any way. The original article has been updated.

