# Corrigendum: Comprehensive pan-cancer analysis of the prognostic and immunological roles of the METTL3/lncRNA-SNHG1/miRNA-140-3p/UBE2C axis

**DOI:** 10.3389/fcell.2024.1392532

**Published:** 2024-04-05

**Authors:** Xiulin Jiang, Yixiao Yuan, Lin Tang, Juan Wang, Qianqian Liu, Xiaolan Zou, Lincan Duan

**Affiliations:** ^1^ Key Laboratory of Animal Models and Human Disease Mechanisms of Chinese Academy of Sciences and Yunnan Province, Kunming Institute of Zoology, Kunming, China; ^2^ Department of Thoracic Surgery, The Third Affiliated Hospital of Kunming Medical University, Kunming, China

**Keywords:** epigenetics, SNHG1, miRNA-140-3p, UBE2C, pan-cancer, prognosis, immunotherapy, drug sensitivity

In the published article, there was an error in Figure 9E as published. Due to negligence and lack of careful inspection, and this part of the sequence is very similar. Therefore, some base mapping errors occurred, the inaccurate WT sequence for SNHG1 is:UCUUUAUCUUGAGCUGUGGUA (In Figure 9E). The corrected WT sequence for SNHG1 is: AUU​UUU​CUA​CUG​CUC​GUG​GUA. The Figure 9E caption was correct. The corrected Figure 9E appear below.

**FIGURE 9:**
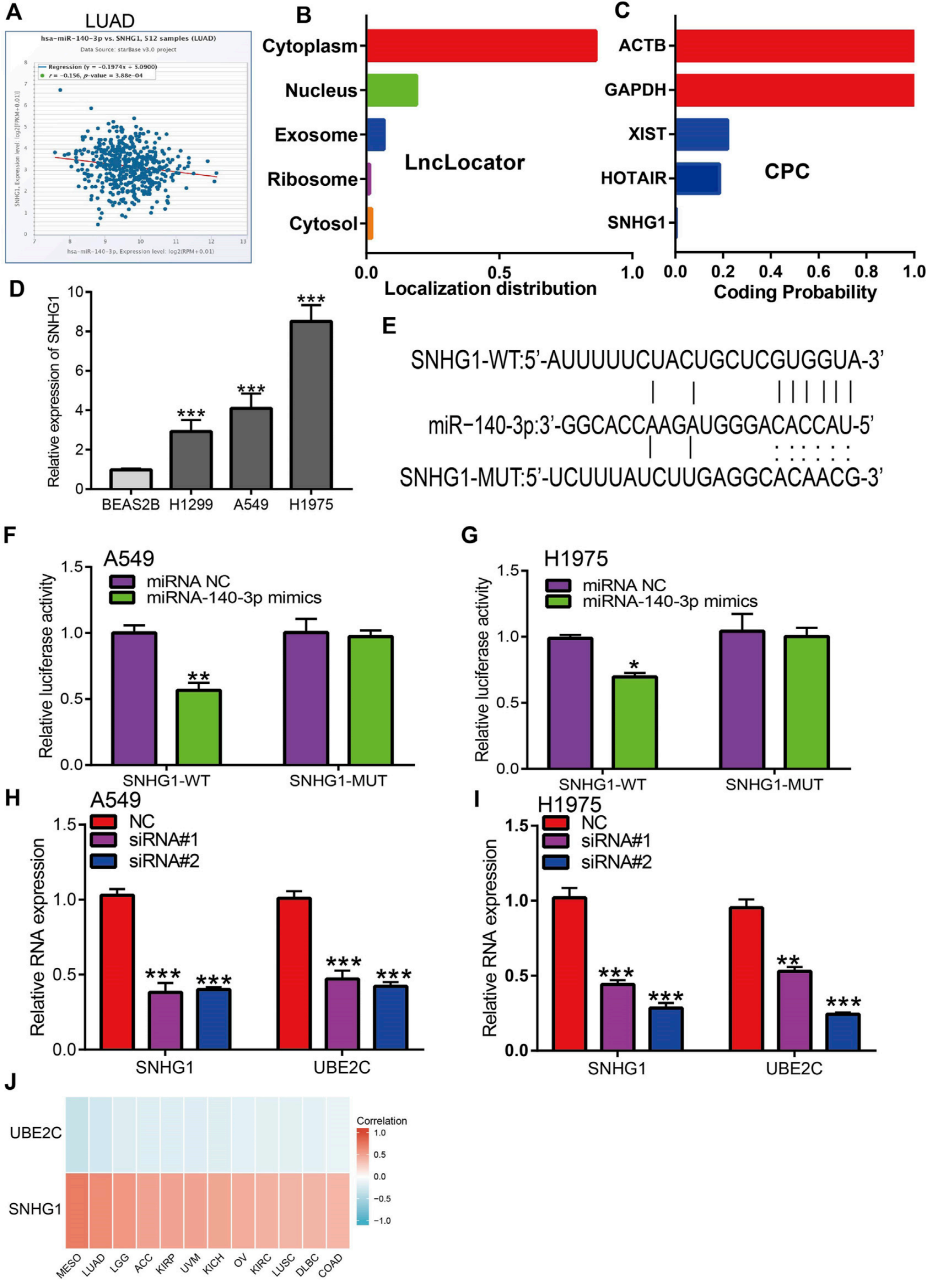
Analysis of upstream lncRNA of miRNA-140-3p in pan-cancer. **(A)** The correlation between the SNHG1 and miRNA-140-3p in LUAD analysis by Starbase. **(B)** The subcellular localization of SNHG1 examined by lncLocator. **(C)** The coding potential of SNHG1 analysis by coding the potential calculator. **(D)** The expression of SNHG1 in NSCLC cell lines examined by qRT-PCR assay. **(E)** The target sites between miRNA-140-3p and SNHG1 was predicted by Starbase. **(F,G)** Relative luciferase activities of wild-type (WT) and mutated (MUT) SNHG1 reporter plasmid in A549 and H1975 cells co-transfected with miR-140-3p mimics examined by luciferase reporter assay. **(H,I)** The expression of UBE2C after depletion of SNHG1 in NSCLC cell lines examined by qRT-PCR assay. **(J)** The correlation between miRNA-140-3p and UBE2C, SNHG1 in NSCLC examined by Starbase. ^*^ *p* < 0.05, ^**^ *p* < 0.01, ^***^ *p* < 0.001.

In the published article, there was an error in the **Materials and methods**, Quantitative Real-Time PCR section: primer sequences: SNHG1-F: AGC​ATC​CAC​GAG​CAA​GAG​AC, SNHG1-R: GAT​GCT​ACT​AGT​GTG​GCG​GG.

A correction has been made to primer sequences: SNHG1-F: GCA​TCT​CAT​AAT​CTA​TCC​TGG, SNHG1- R:CCTAGTTTTCCTCAAACTCCT. This sentence previously stated:

“SNHG1-F: AGC​ATC​CAC​GAG​CAA​GAG​AC, SNHG1-R: GAT​GCT​ACT​AGT​GTG​GCG​GG”

The corrected sentence appears below:

“SNHG1-QPCR-F-GCATCTCATAATCTATCCTGG, SNHG1-QPCR-R-CCTAGTTTTCCTCAAACTCCT”

The authors apologize for these errors and state that this does not change the scientific conclusions of the article in any way. The original article has been updated.

