# Supplementary material for: Comprehensive Pan-Cancer Analysis of the Prognostic and Immunological Roles of the METTL3/lncRNA-SNHG1/miRNA-140-3p/UBE2C Axis
Source: Front Cell Dev Biol. 2021 Nov 10;9:765772. doi: 10.3389/fcell.2021.765772 (PMC8631498; doi:10.3389/fcell.2021.765772)
Supplement: Supplementary file 1 [file DataSheet1.docx]

Supplementary Material

**Supplementary Figures**


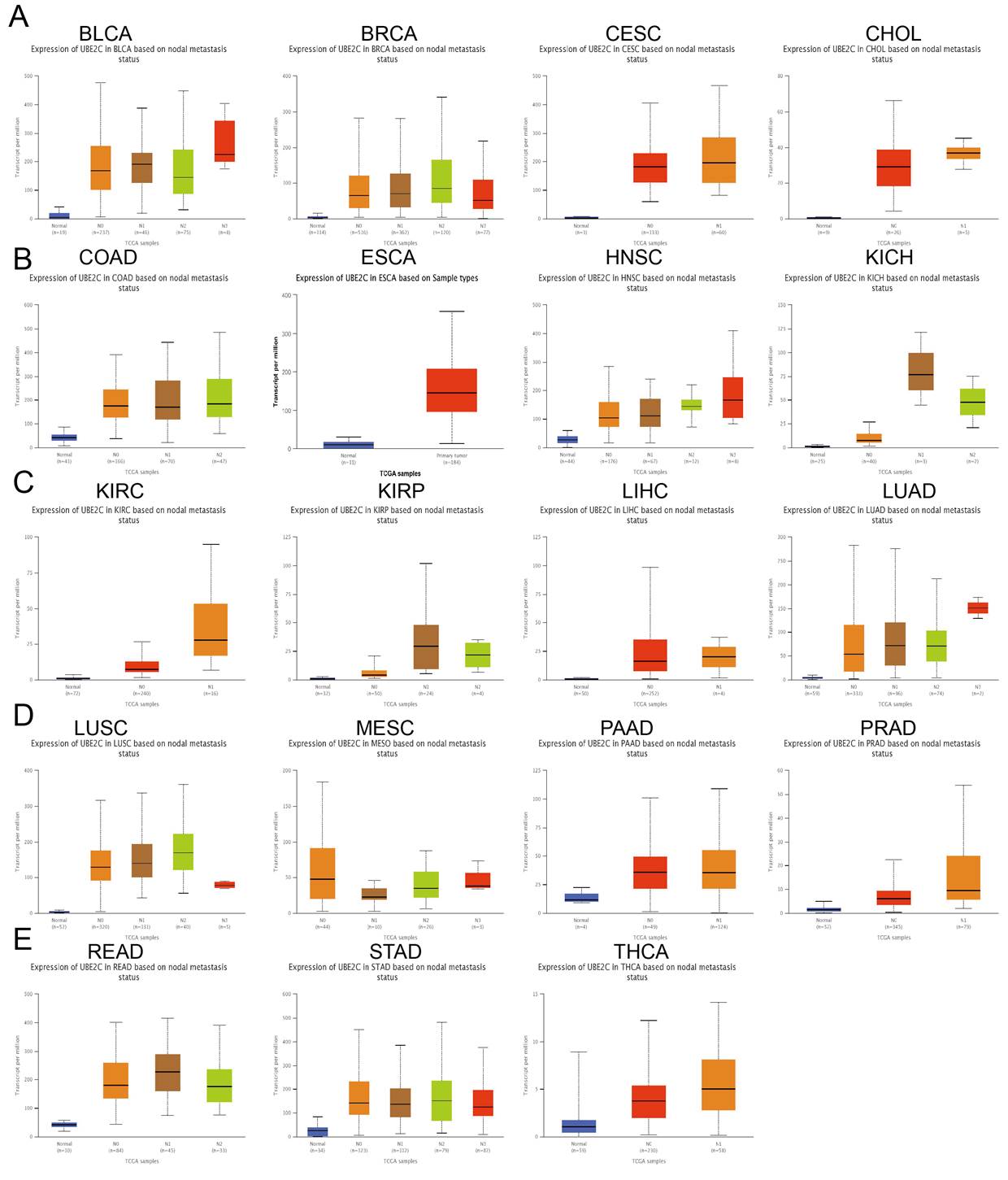


**Supplementary FIGURE 1⎜ Association between the UBE2C expression and Lymph node metastasis of pan-cancer**

The correlation between the BUE2C expression and Lymph node metastasis in BLCA, BRCA, CESC and CHOL (A), COAD, ESCA, HNSC and KICH (B), KIRC, KIRP , LIHC and LUAD (C), LUSC, MESC, PAAD and PRAD (D), READ, STAD and THCA (E) analysis by the UALCAN database.


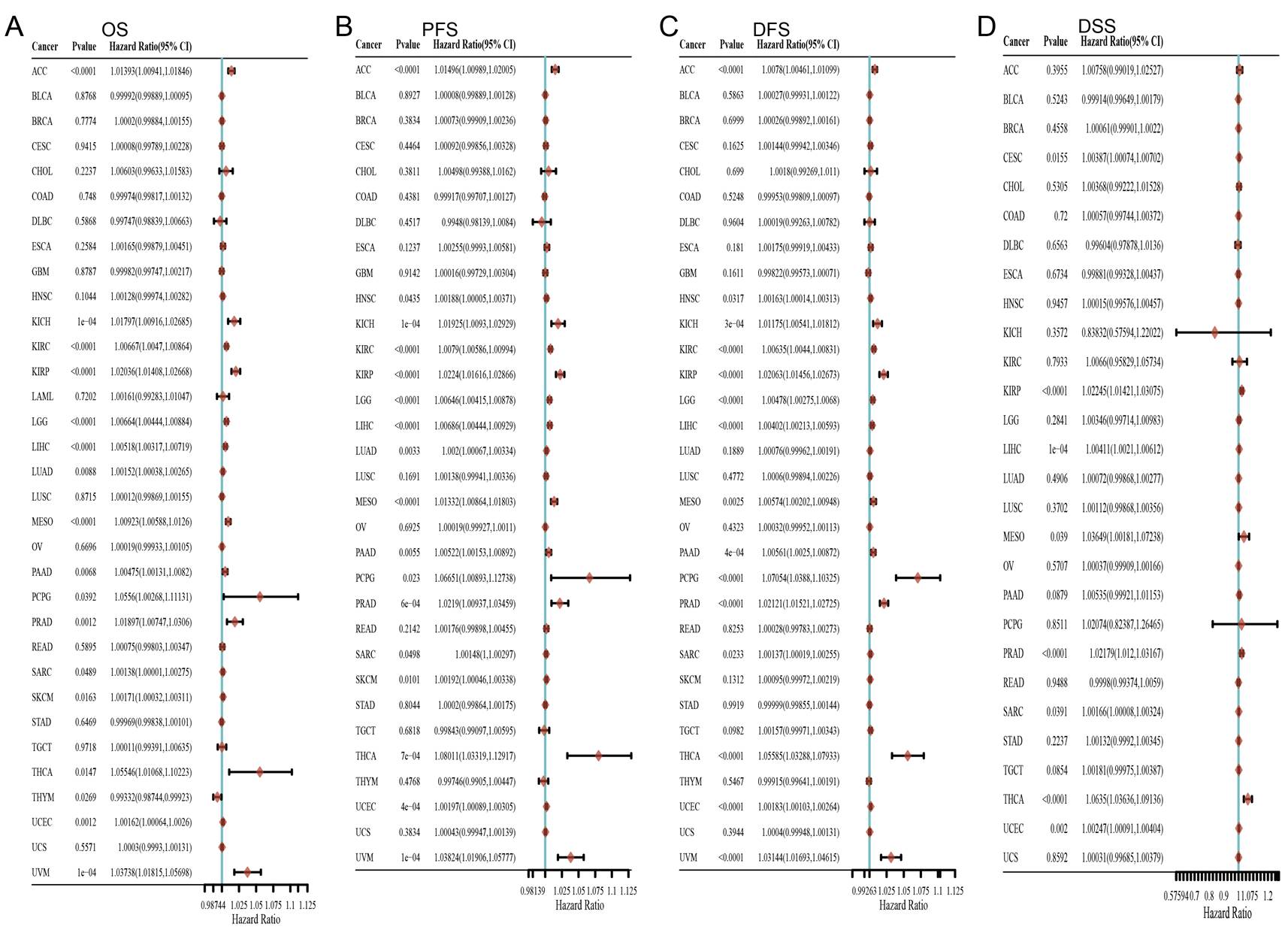


**Supplementary FIGURE 2 ⎜ Association between the UBE2C expression and the OS, PFS, DFS and DSS of cancer patients.** (A) A forest plot of hazard ratios shown that the OS of SNX20 in 33 types of tumors. (B) A forest plot of hazard ratios shown that the PFS of UBE2C in 33 types of tumors. (C) A forest plot of hazard ratios shown that the DFS of UBE2C in 33 types of tumors. (D) A forest plot of hazard ratios shown that the DSS ofUBE2C in 33 types of tumors.


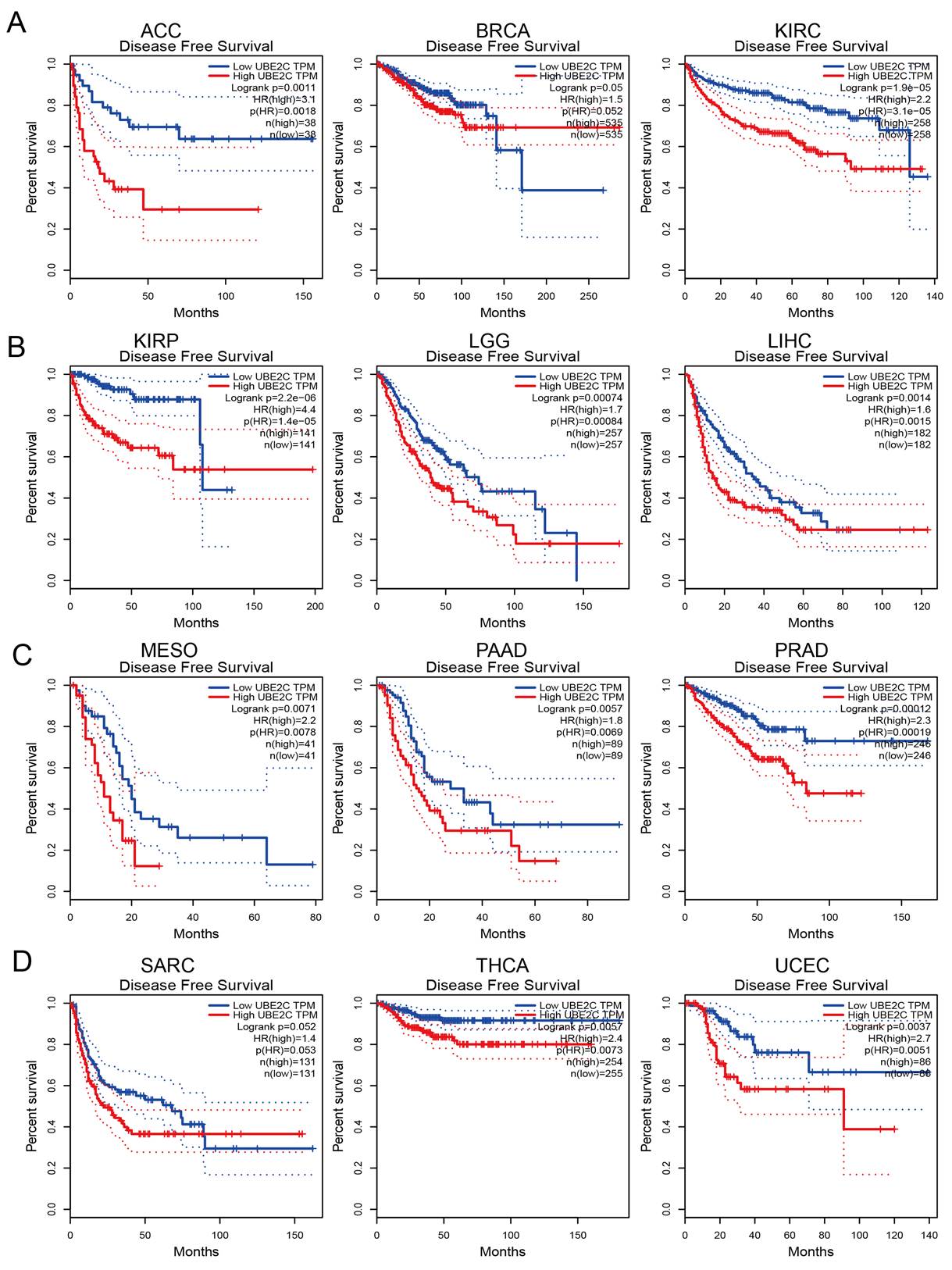


**Supplementary FIGURE 3 ⎜ Analysis the disease free survival for UBE2C in human cancers.**

(A)The disease free survival for UBE2C in ACC, BRCA and KIRC, (B) KIRP, LGG and LIHC, (C) MESO, PAAD and PRAD, (D)SARC, THCA and UCEC analysis by GEPIA database.


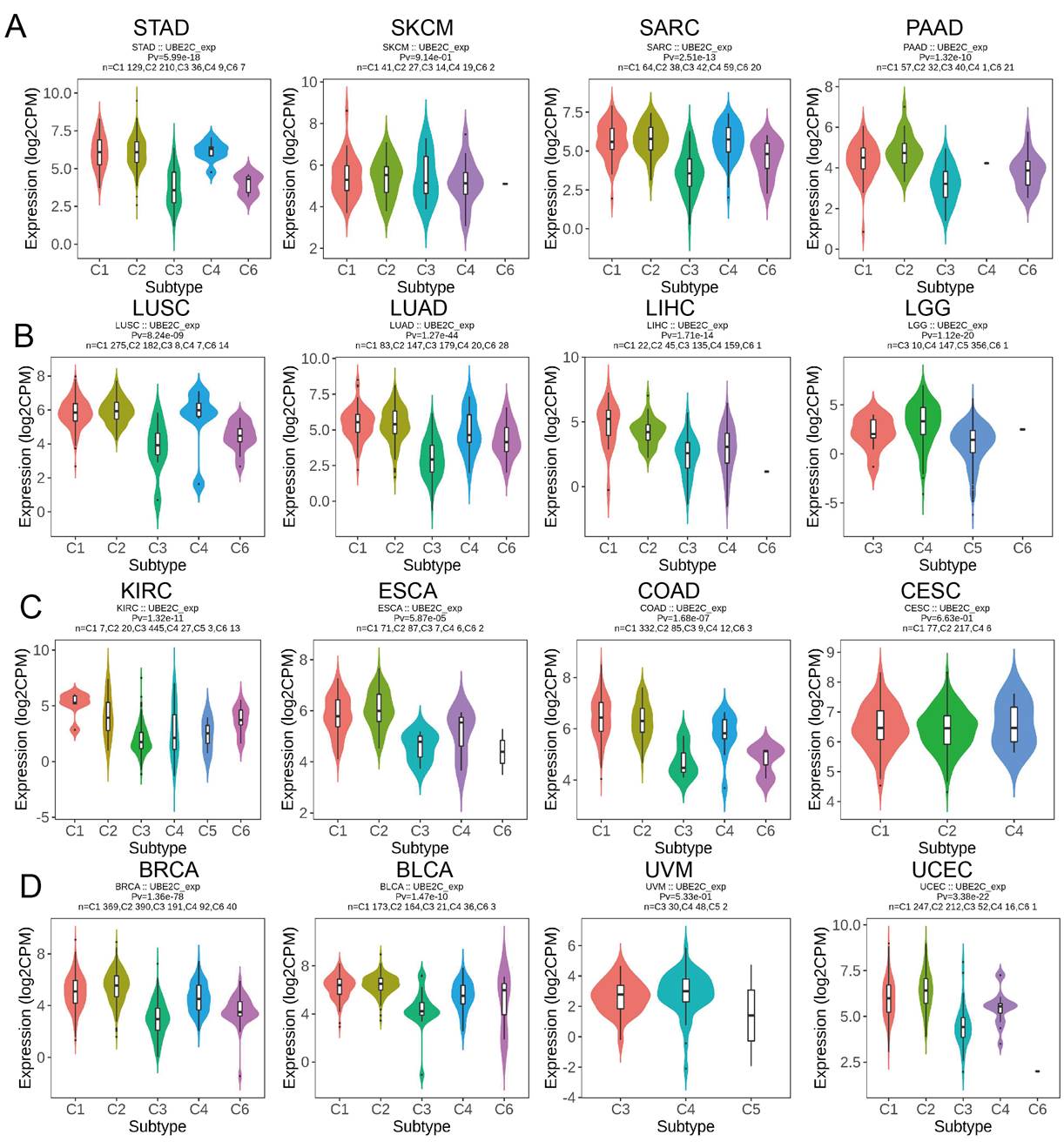


**Supplementary FIGURE 4⎜ The expression of UBE2C in immune subtypes of pan-cancer.**

The expression of UBE2C in STAD, SKCM, SARC and PAAD (A), LUSC, LUAD, LIHC and LGG (B), KIRC, ESCA, COAD and CESC (C), BRCA, BLCA, UVM and UCEC (D) immune subtypes analysis by the TISIDB database.


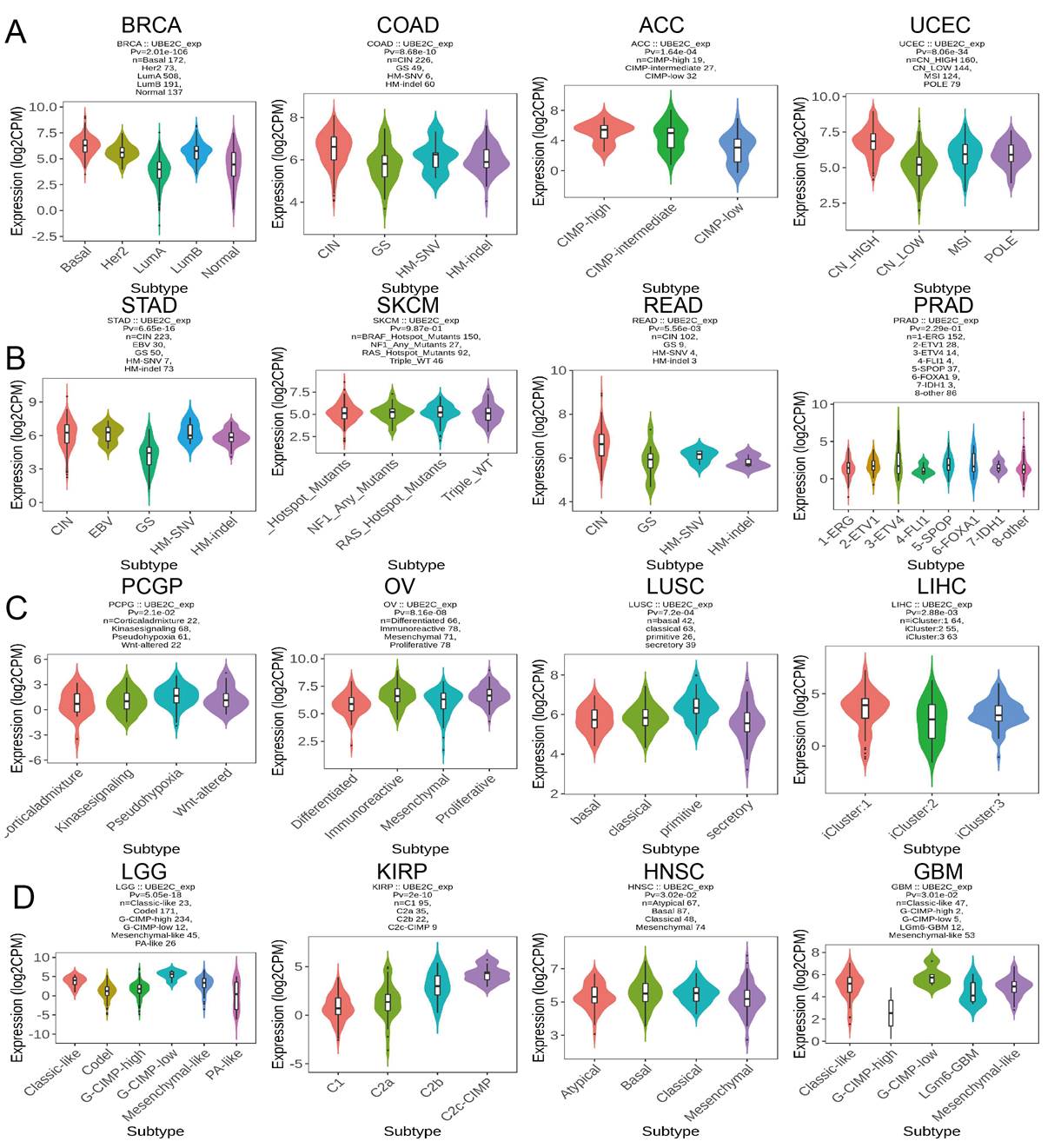


**Supplementary FIGURE 5 ⎜ The expression of UBE2C in molecular subtypes of pan-cancer.**

The expression of UBE2C in BRCA, COAD, ACC and UCEC (A), STAD, SKCM, READ and PRAD (B), PCPG, OV, LUSC and LIHC (C), LGG, KIRP, HNSC and GBM (D) molecular subtypes analysis by the TISIDB database.


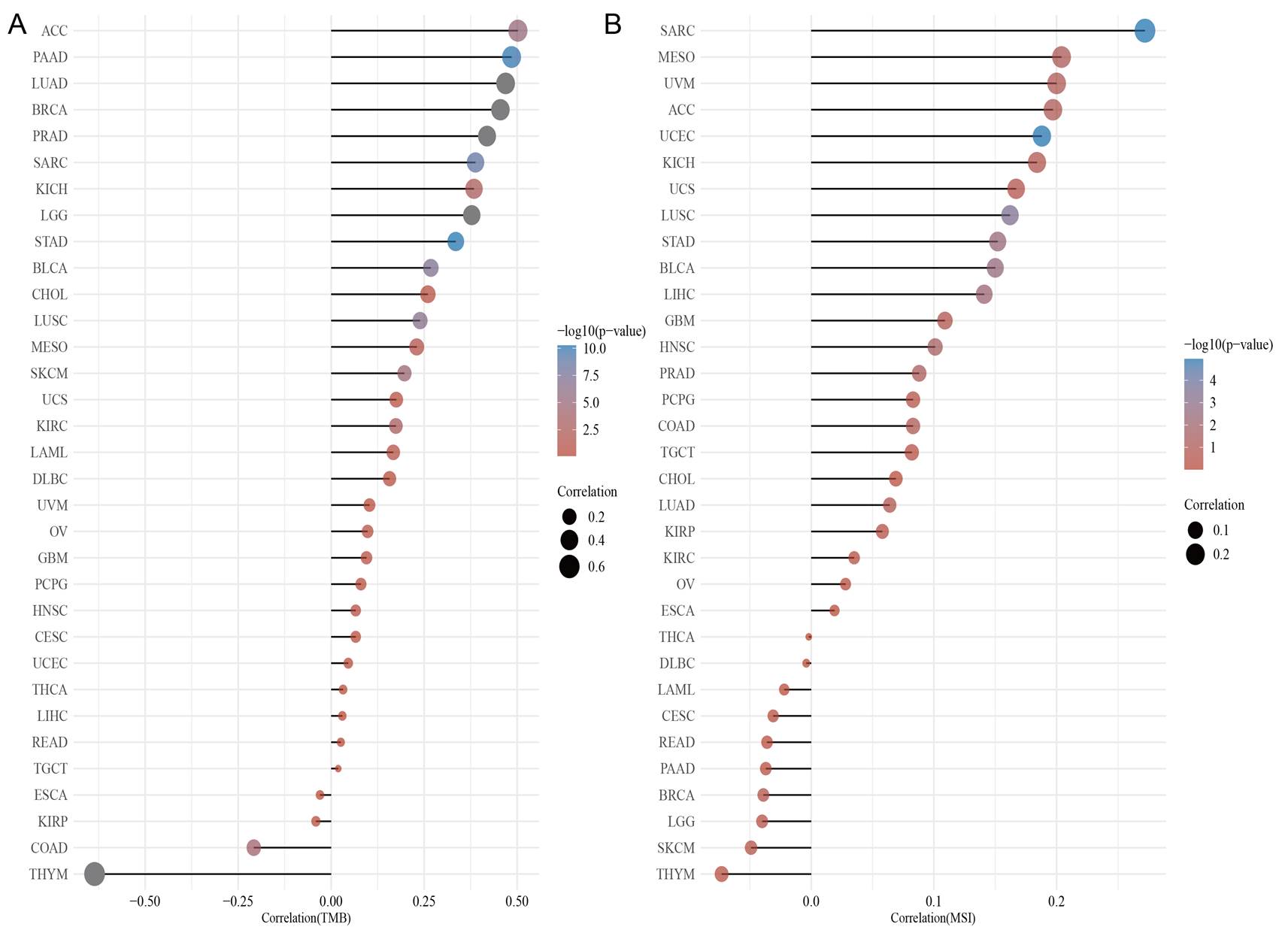


**Supplementary FIGURE 6 ⎜ The correlation between the UBE2C expression and TMB, MSI in pan-cancer.**

The correlation between the UBE2C expression and TMB (A), MSI (B).


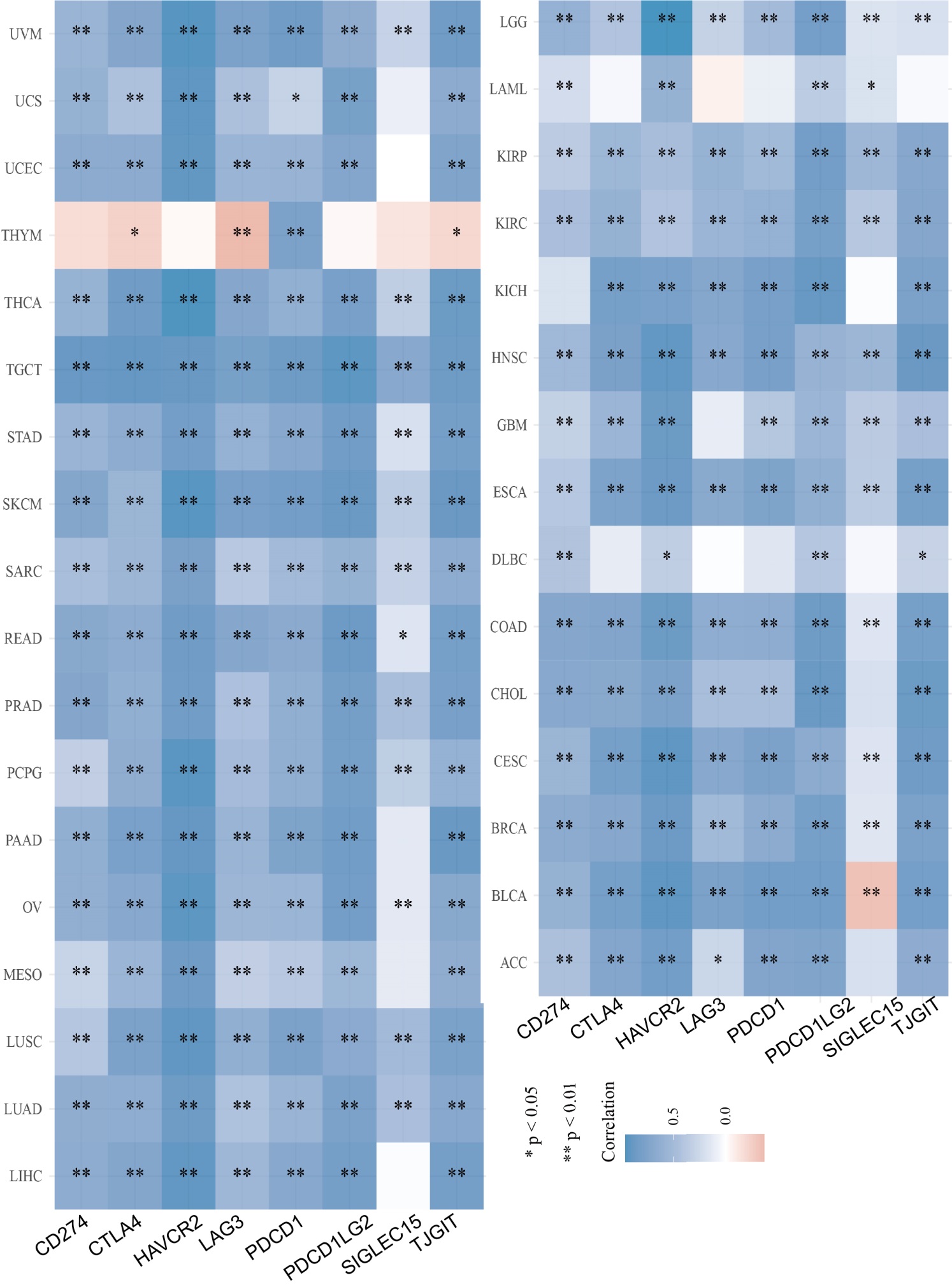


**Supplementary FIGURE 7 ⎜ Analysis the correlation between the UBE2C expression and immune check points related gene.** Analysis the correlation between the UBE2C expression and immune check points related gene in pan-cancer analysis by TIMER database.


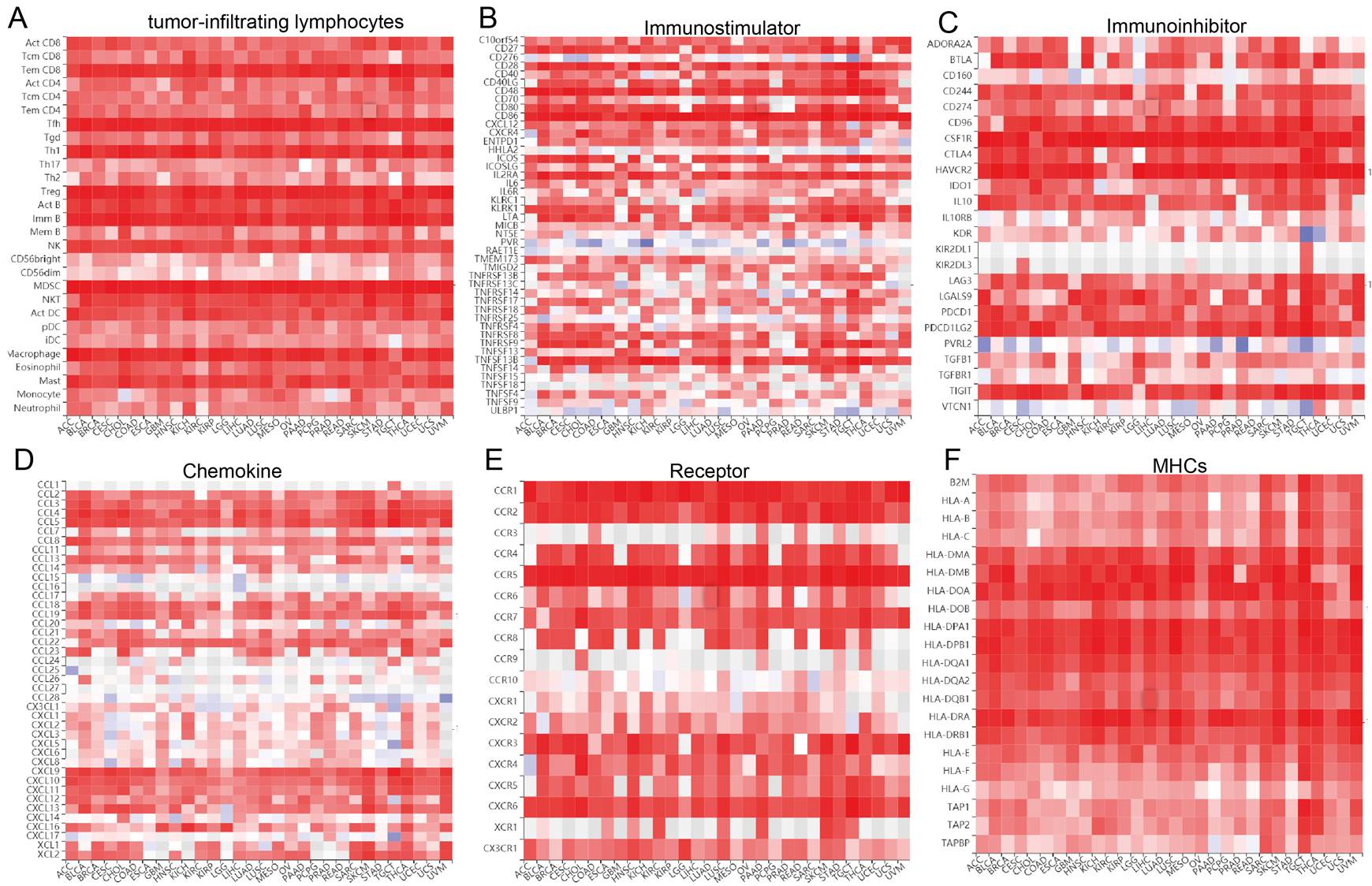


**Supplementary FIGURE 8 ⎜ Analysis the correlation between the UBE2C expression and diverse immune regulator.** (A)The correlation between the UBE2C expression and 28 tumor infiltrating lymphocytes analysis in pan-cancer by the TISIDB database. (B) The correlation between the UBE2C expression and 45 immune-stimulator in pan-cancer analysis by the TISIDB database. (C)The correlation between the UBE2C expression and 24 immune-inhibitor in pan-cancer analysis by the TISIDB database. (D) The correlation between the UBE2C expression and 41 chemokine in pan-cancer analysis by the TISIDB database. (E) The correlation between the UBE2C expression and 18 receptor in pan-cancer analysis by the TISIDB database. (F) The correlation between the UBE2C expression and 21 MHCs in pan-cancer analysis by the TISIDB database.


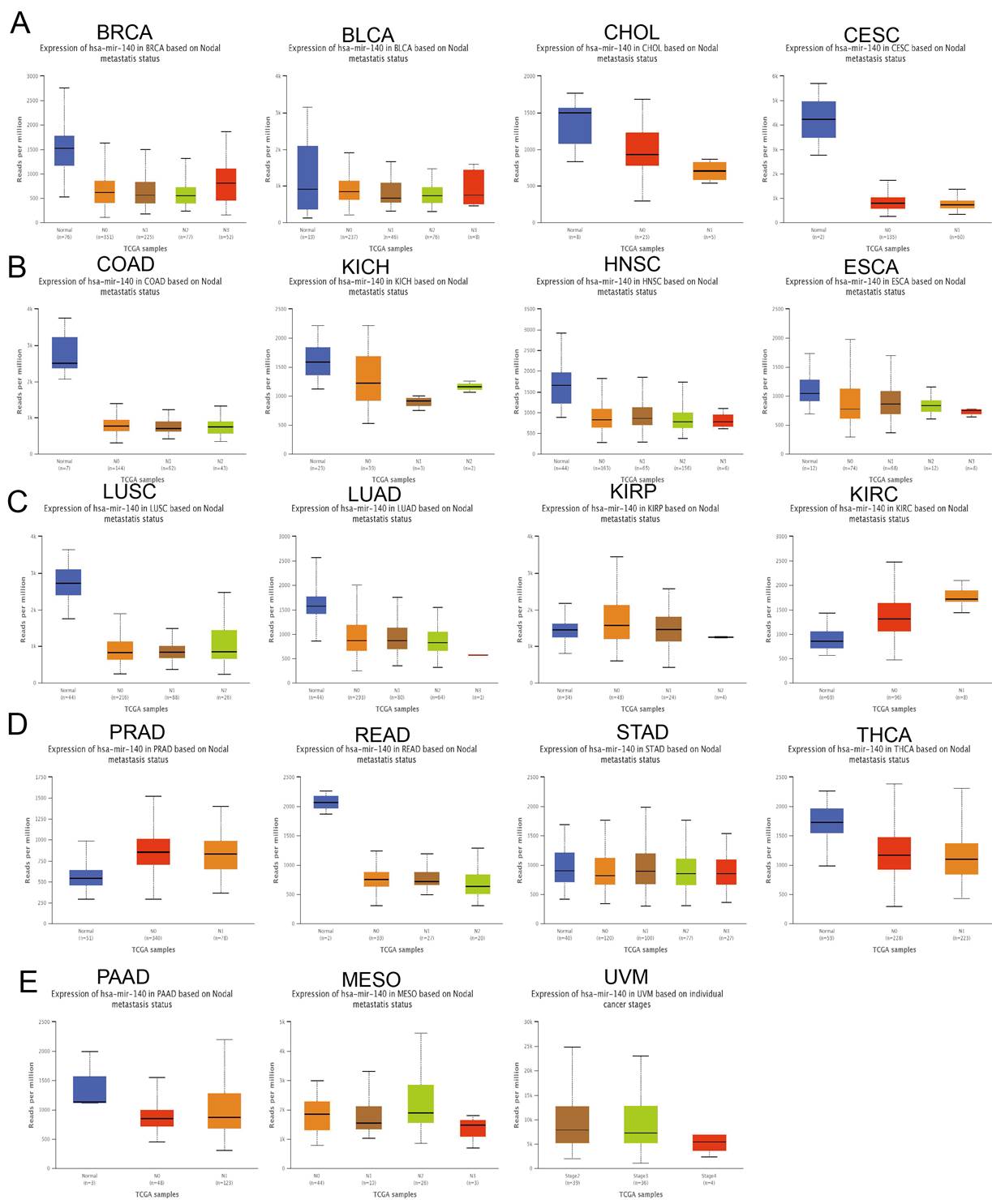


**Supplementary FIGURE 9⎜ The correlation between the miRNA-140-3p expression and the Lymph node metastasis of pan-cancer**

The correlation between the miRNA-140-3p expression and the Lymph node metastasis in BRCA, BLCA, CHOL and CESC (A), COAD, KICH, HNSC and ESCA (B), LUSC, LUAD, KIRP and KIRC (C), PRAD, READ, STAD and THCA (D), PAAD, MESO and UVM (E) analysis by the starbase database.


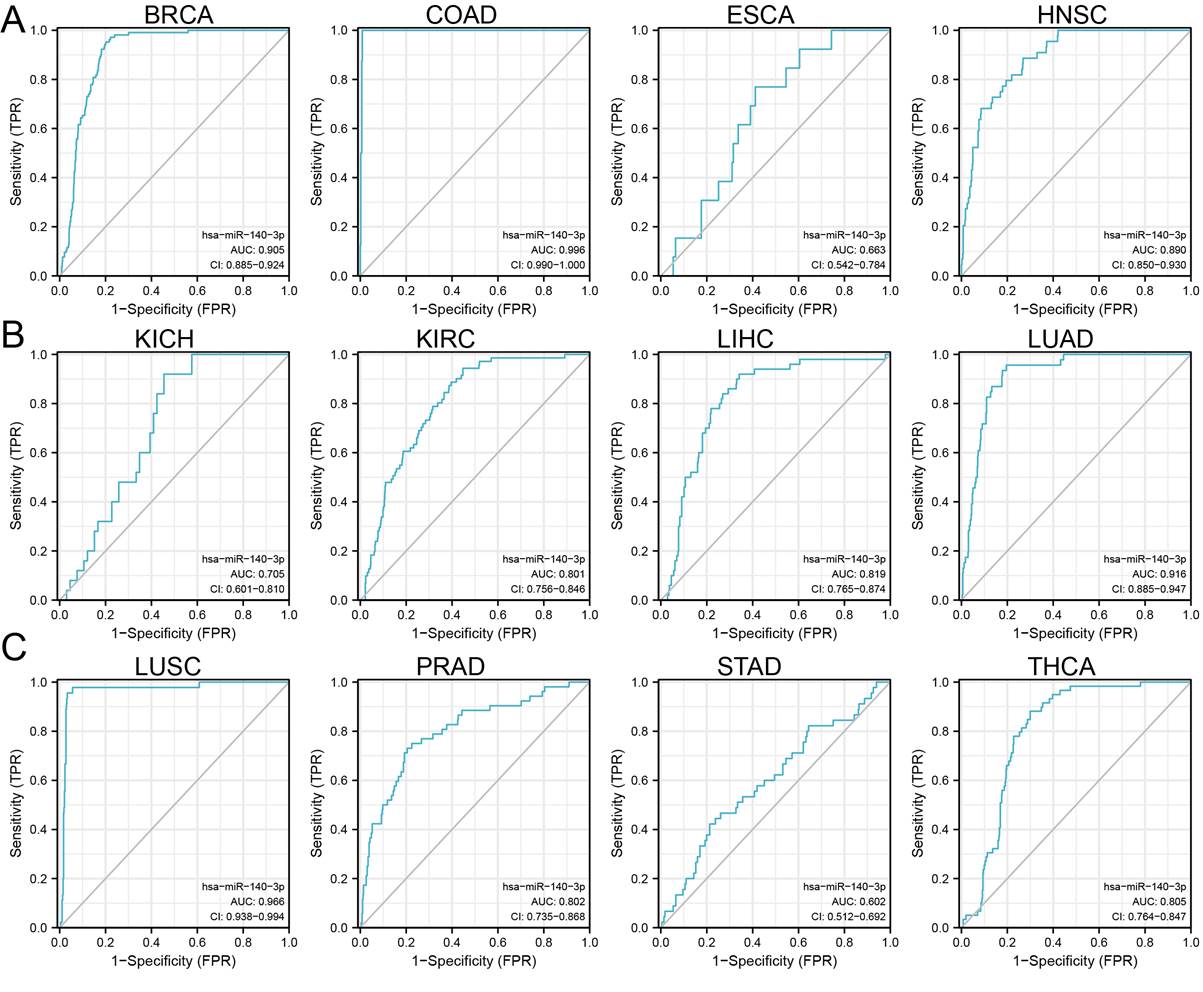


**Supplementary FIGURE 10⎜ ROC curve analyses and AUC values for miRNA-140-3p in diverse cancer.**

(A)ROC curve analyses and AUC values for miRNA-140-3p in diverse cancer.


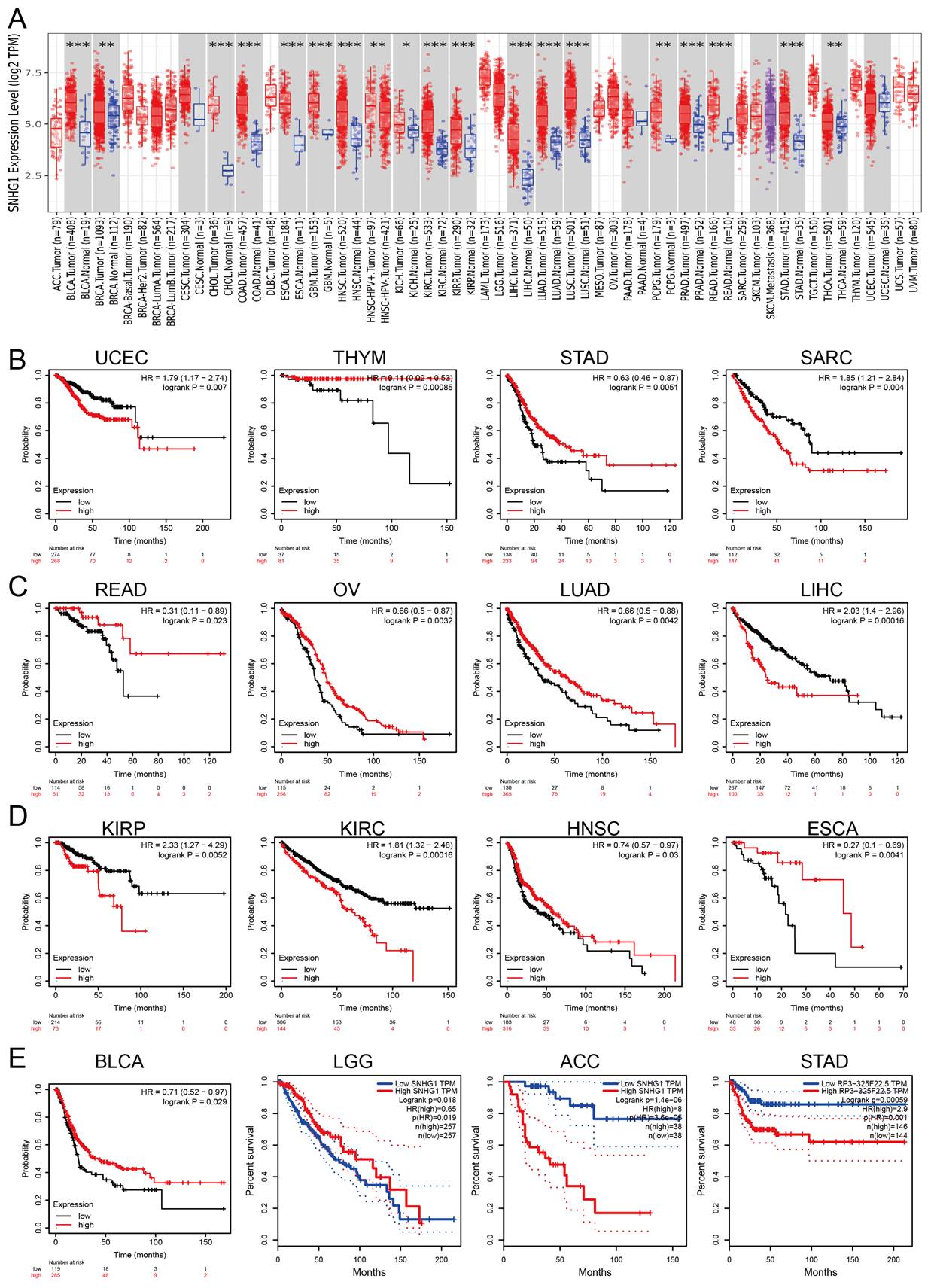


**Supplementary FIGURE11 ⎜ Analysis the expression and prognosis of SNHG1 in pan-cancer.** (A) The expression of SNHG1 in pan-cancer analysis by TIMER database. (B) The prognosis of SNHG1 in UCEC, THYM, STAD and SARC analysis by kmplot database. (C) The prognosis of SNHG1 in READ, OV, LUAD and LIHC analysis by kmplot database. (D) The prognosis of SNHG1 in KIRP, KIRC, HNSC and ESCA analysis by kmplot database. (E) The prognosis of SNHG1 in BLCA, LGG, ACC and STAD analysis by kmplot and GEPIA databases.


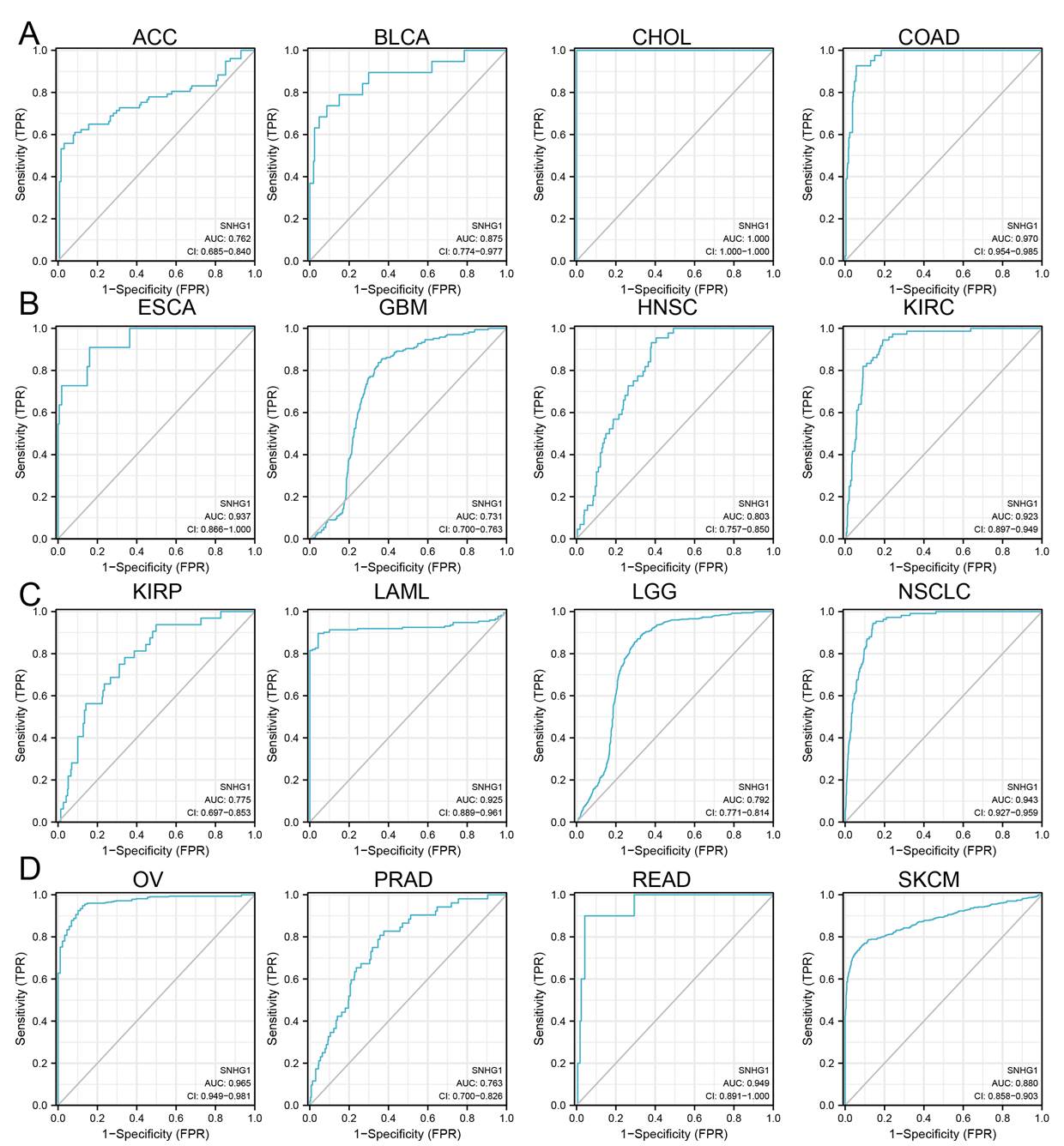


**Supplementary FIGURE 12⎜ ROC curve analyses and AUC values for SNHG1 in diverse cancer.**

(A-D)ROC curve analyses and AUC values for SNHG1 in diverse cancer.

**Supplementary Table 1. Correlation analysis between UBE2C and diverse immune cells related gene marker in BLCA, BRCA, CESC, COAD, HNSC, KIRC, KIRP, LGG and LIHC analysis by TIMER.**

| Gene markers | BLCA | BRCA | CESC | COAD | HNSC | KIRC | KIRP | LGG | LIHC |  |
| --- | --- | --- | --- | --- | --- | --- | --- | --- | --- | --- |
|  | Cor | Cor | Cor | Cor | Cor | Cor | Cor | Cor | Cor | P |
| CD19 | 0.583 | 0.545 | 0.588 | 0.573 | 0.566 | 0.424 | 0.443 | 0.420 | 0.560 | *** |
| CD79A | 0.684 | 0.555 | 0.629 | 0.652 | 0.575 | 0.471 | 0.534 | 0.382 | 0.664 | *** |
| CD3D | 0.782 | 0.736 | 0.783 | 0.734 | 0.769 | 0.650 | 0.664 | 0.427 | 0.716 | *** |
| CD3E | 0.867 | 0.758 | 0.851 | 0.810 | 0.873 | 0.672 | 0.698 | 0.461 | 0.815 | *** |
| CD2 | 0.866 | 0.787 | 0.854 | 0.789 | 0.863 | 0.711 | 0.688 | 0.467 | 0.796 | *** |
| CD8A | 0.774 | 0.716 | 0.778 | 0.708 | 0.781 | 0.633 | 0.666 | 0.164 | 0.757 | *** |
| CD8B | 0.627 | 0.617 | 0.488 | 0.404 | 0.740 | 0.594 | 0.628 | 0.197 | 0.683 | *** |
| CD86 | 0.893 | 0.905 | 0.902 | 0.892 | 0.843 | 0.904 | 0.924 | 0.945 | 0.946 | *** |
| CSF1R | 0.912 | 0.851 | 0.903 | 0.911 | 0.931 | 0.853 | 0.894 | 0.908 | 0.907 | *** |
| CCL2 | 0.701 | 0.556 | 0.407 | 0.682 | 0.630 | 0.060 | 0.440 | 0.523 | 0.602 | *** |
| CD68 | 0.651 | 0.858 | 0.560 | 0.717 | 0.562 | 0.700 | 0.586 | 0.914 | 0.711 | *** |
| IL10 | 0.762 | 0.750 | 0.564 | 0.724 | 0.655 | 0.712 | 0.770 | 0.678 | 0.749 | *** |
| IRF5 | 0.345 | 0.521 | 0.288 | 0.371 | 0.387 | 0.457 | 0.192 | 0.882 | 0.315 | *** |
| CD163 | 0.894 | 0.806 | 0.805 | 0.876 | 0.795 | 0.8 | 0.888 | 0.867 | 0.77 | *** |
| VSIG4 | 0.89 | 0.678 | 0.696 | 0.826 | 0.726 | 0.771 | 0.849 | 0.502 | 0.825 | *** |
| MS4A4A | 0.912 | 0.827 | 0.834 | 0.859 | 0.827 | 0.805 | 0.891 | 0.74 | 0.853 | *** |
| ITGAM | 0.869 | 0.72 | 0.675 | 0.802 | 0.732 | 0.823 | 0.856 | 0.893 | 0.656 | *** |
| CCR7 | 0.182 | 0.667 | 0.675 | 0.734 | 0.756 | 0.605 | 0.662 | 0.302 | 0.664 | *** |
| KIR2DL1 | 0.44 | 0.391 | 0.337 | 0.308 | 0.337 | 0.096 | 0.172 | 0.004 | 0.141 | *** |
| KIR2DL3 | 0.537 | 0.42 | 0.524 | 0.393 | 0.471 | 0.116 | 0.272 | 0.138 | 0.326 | *** |
| KIR2DL4 | 0.592 | 0.497 | 0.468 | 0.424 | 0.515 | 0.262 | 0.301 | 0.471 | 0.47 | *** |
| KIR3DL1 | 0.479 | 0.469 | 0.458 | 0.388 | 0.446 | 0.106 | 0.298 | -0.008 | 0.268 | *** |
| KIR3DL2 | 0.489 | 0.505 | 0.549 | 0.451 | 0.597 | 0.171 | 0.339 | 0.17 | 0.38 | *** |
| KIR2DS4 | 0.474 | 0.361 | 0.428 | 0.318 | 0.338 | 0.073 | 0.231 | 0.154 | 0.227 | *** |
| HLA-DPB1 | 0.772 | 0.779 | 0.731 | 0.844 | 0.872 | 0.834 | 0.892 | 0.676 | 0.86 | *** |
| HLA-DQB1 | 0.878 | 0.634 | 0.578 | 0.576 | 0.699 | 0.524 | 0.7 | 0.527 | 0.74 | *** |
| HLA-DRA | 0.84 | 0.875 | 0.67 | 0.791 | 0.853 | 0.875 | 0.903 | 0.741 | 0.849 | *** |
| HLA-DPA1 | 0.841 | 0.85 | 0.693 | 0.835 | 0.864 | 0.837 | 0.883 | 0.691 | 0.872 | *** |
| CD1C | 0.541 | 0.567 | 0.493 | 0.626 | 0.647 | 0.423 | 0.638 | 0.408 | 0.604 | *** |
| NRP1 | 0.545 | 0.451 | 0.196 | 0.735 | 0.551 | 0.258 | 0.226 | 0.205 | 0.459 | *** |
| ITGAX | 0.873 | 0.851 | 0.799 | 0.844 | 0.792 | 0.641 | 0.77 | 0.754 | 0.826 | *** |
| TBX21 | 0.816 | 0.737 | 0.816 | 0.730 | 0.797 | 0.420 | 0.633 | 0.241 | 0.694 | *** |
| STAT4 | 0.765 | 0.729 | 0.643 | 0.735 | 0.699 | 0.525 | 0.514 | -0.226 | 0.517 | *** |
| STAT1 | 0.622 | 0.599 | 0.618 | 0.640 | 0.540 | 0.762 | 0.331 | 0.412 | 0.621 | *** |
| TNF | 0.455 | 0.387 | 0.182 | 0.517 | 0.231 | 0.450 | 0.382 | 0.388 | 0.684 | *** |
| IFNG | 0.660 | 0.644 | 0.683 | 0.528 | 0.602 | 0.573 | 0.337 | 0.243 | 0.591 | *** |
| HAVCR2 | 0.932 | 0.881 | 0.933 | 0.878 | 0.918 | 0.416 | 0.479 | 0.960 | 0.909 | *** |
| CXCR3 | 0.864 | 0.751 | 0.815 | 0.342 | 0.864 | 0.655 | 0.651 | 0.492 | 0.742 | *** |
| BHLHE40 | 0.225 | -0.034 | 0.149 | 0.261 | 0.255 | 0.025 | 0.222 | 0.111 | 0.215 | *** |
| CD4 | 0.888 | 0.956 | 0.933 | 0.952 | 0.970 | 0.918 | 0.949 | 0.920 | 0.774 | *** |
| STAT6 | -0.120 | 0.163 | 0.223 | 0.192 | 0.348 | 0.261 | 0.214 | 0.522 | 0.203 | *** |
| STAT5A | 0.497 | 0.379 | 0.514 | 0.482 | 0.650 | 0.747 | 0.666 | 0.761 | 0.632 | *** |
| FOXP3 | 0.795 | 0.713 | 0.792 | 0.804 | 0.885 | 0.576 | 0.420 | -0.170 | 0.430 | *** |
| CCR8 | 0.713 | 0.725 | 0.748 | 0.756 | 0.819 | 0.678 | 0.499 | 0.134 | 0.704 | *** |
| TGFB1 | 0.349 | 0.472 | 0.228 | 0.737 | 0.084 | 0.251 | 0.402 | 0.804 | 0.607 | *** |
| TNFRSF9 | 0.807 | 0.813 | 0.752 | 0.804 | 0.834 | 0.702 | 0.525 | 0.256 | 0.665 | *** |
| FGFBP2 | 0.462 | 0.284 | 0.165 | 0.458 | 0.055 | 0.060 | 0.221 | 0.347 | 0.202 | *** |
| FCGR3A | 0.886 | 0.742 | 0.811 | 0.817 | 0.818 | 0.870 | 0.813 | 0.825 | 0.817 | *** |
| CCR7 | 0.182 | 0.667 | 0.675 | 0.734 | 0.756 | 0.605 | 0.662 | 0.302 | 0.664 | *** |
| SELL | 0.405 | 0.694 | 0.680 | 0.804 | 0.823 | 0.754 | 0.755 | 0.108 | 0.743 | *** |
| GZMK | 0.837 | 0.721 | 0.789 | 0.772 | 0.795 | 0.627 | 0.710 | 0.446 | 0.739 | *** |
| CD69 | 0.656 | 0.707 | 0.685 | 0.747 | 0.768 | 0.648 | 0.690 | 0.685 | 0.757 | *** |
| CXCR6 | 0.791 | 0.794 | 0.808 | 0.740 | 0.848 | 0.754 | 0.724 | 0.471 | 0.790 | *** |
| MYADM | 0.545 | 0.086 | -0.028 | 0.353 | 0.155 | 0.085 | 0.259 | 0.431 | 0.461 | *** |
| IL7R | 0.761 | 0.770 | 0.525 | 0.742 | 0.525 | 0.682 | 0.632 | 0.431 | 0.702 | *** |
| HAVCR2 | 0.932 | 0.881 | 0.933 | 0.878 | 0.918 | 0.416 | 0.479 | 0.960 | 0.909 | *** |
| LAG3 | 0.787 | 0.552 | 0.704 | 0.712 | 0.700 | 0.567 | 0.546 | 0.283 | 0.559 | *** |
| CXCL13 | 0.753 | 0.498 | 0.704 | 0.673 | 0.681 | 0.466 | 0.306 | -0.156 | 0.437 | *** |
| LAYN | 0.581 | 0.334 | 0.214 | 0.658 | 0.193 | 0.087 | 0.130 | -0.139 | 0.553 | *** |
|  |  |  |  |  |  |  |  |  |  |  |

**Supplementary Table2. Correlation analysis between UBE2C and diverse immune cells related gene marker in LUAD, LUSC, OV, PRAD, SKCM, STAD, THCA and UCEC analysis by TIMER.**

| Description | Gene markers | LUAD | LUSC | OV | PRAD | SKCM | STAD | THCA | UCEC |  |
| --- | --- | --- | --- | --- | --- | --- | --- | --- | --- | --- |
|  |  | Cor | Cor | Cor | Cor | Cor | Cor | Cor | Cor | P |
| B cell | CD19 | 0.459 | 0.608 | 0.177 | 0.513 | 0.683 | 0.596 | 0.687 | 0.463 | *** |
|  | CD79A | 0.429 | 0.63 | 0.458 | 0.595 | 0.716 | 0.624 | 0.762 | 0.649 | *** |
| T cell (general) | CD3D | 0.636 | 0.79 | 0.671 | 0.713 | 0.869 | 0.755 | 0.831 | 0.747 | *** |
|  | CD3E | 0.715 | 0.854 | 0.729 | 0.786 | 0.879 | 0.751 | 0.861 | 0.781 | *** |
|  | CD2 | 0.739 | 0.849 | 0.748 | 0.794 | 0.891 | 0.811 | 0.872 | 0.769 | *** |
| CD8+ T cell | CD8A | 0.59 | 0.755 | 0.65 | 0.746 | 0.851 | 0.724 | 0.64 | 0.708 | *** |
|  | CD8B | 0.478 | 0.575 | 0.489 | 0.458 | 0.825 | 0.533 | 0.62 | 0.454 | *** |
| Monocyte | CD86 | 0.882 | 0.889 | 0.935 | 0.881 | 0.954 | 0.841 | 0.957 | 0.851 | *** |
|  | CSF1R | 0.887 | 0.911 | 0.935 | 0.904 | 0.926 | 0.851 | 0.904 | 0.865 | *** |
| TAM | CCL2 | 0.497 | 0.594 | 0.582 | 0.459 | 0.691 | 0.476 | 0.612 | 0.454 | *** |
|  | CD68 | 0.798 | 0.756 | 0.938 | 0.842 | 0.605 | 0.61 | 0.91 | 0.784 | *** |
|  | IL10 | 0.678 | 0.667 | 0.578 | 0.663 | 0.746 | 0.668 | 0.763 | 0.297 | *** |
| M1 | IRF5 | 0.602 | 0.23 | 0.451 | 0.58 | 0.718 | 0.472 | 0.613 | 0.385 | *** |
| M2 | CD163 | 0.820 | 0.856 | 0.91 | 0.78 | 0.827 | 0.797 | 0.824 | 0.73 | *** |
|  | VSIG4 | 0.786 | 0.819 | 0.839 | 0.79 | 0.795 | 0.694 | 0.85 | 0.762 | *** |
|  | MS4A4A | 0.821 | 0.851 | 0.867 | 0.776 | 0.875 | 0.814 | 0.881 | 0.806 | *** |
| Neutrophils | CEACAM8 | 0.327 | 0.144 | -0.014 | 0.079 | 0.325 | 0.058 | 0.317 | 0.052 | *** |
|  | ITGAM | 0.833 | 0.807 | 0.913 | 0.823 | 0.818 | 0.773 | 0.897 | 0.778 | *** |
|  | CCR7 | 0.661 | 0.755 | 0.674 | 0.758 | 0.775 | 0.755 | 0.842 | 0.688 | *** |
| Natural killer cell | KIR2DL1 | 0.2 | 0.310 | 0.199 | 0.143 | 0.402 | 0.338 | 0.163 | 0.354 | *** |
|  | KIR2DL3 | 0.307 | 0.398 | 0.275 | 0.152 | 0.571 | 0.308 | 0.323 | 0.346 | *** |
|  | KIR2DL4 | 0.267 | 0.432 | 0.497 | 0.283 | 0.672 | 0.335 | 0.332 | 0.534 | *** |
|  | KIR3DL1 | 0.253 | 0.511 | 0.336 | 0.293 | 0.534 | 0.36 | 0.318 | 0.426 | *** |
|  | KIR3DL2 | 0.357 | 0.492 | 0.273 | 0.177 | 0.635 | 0.435 | 0.453 | 0.387 | *** |
|  | KIR2DS4 | 0.268 | 0.360 | 0.21 | 0.203 | 0.445 | 0.297 | 0.267 | 0.369 | *** |
| Dendritic cell | HLA-DPB1 | 0.749 | 0.929 | 0.725 | 0.77 | 0.873 | 0.767 | 0.874 | 0.744 | *** |
|  | HLA-DQB1 | 0.548 | 0.712 | 0.483 | 0.628 | 0.807 | 0.572 | 0.607 | 0.544 | *** |
|  | HLA-DRA | 0.746 | 0.903 | 0.664 | 0.86 | 0.894 | 0.736 | 0.882 | 0.675 | *** |
|  | HLA-DPA1 | 0.760 | 0.928 | 0.723 | 0.853 | 0.861 | 0.725 | 0.863 | 0.772 | *** |
|  | CD1C | 0.502 | 0.593 | 0.544 | 0.767 | 0.672 | 0.671 | 0.705 | 0.55 | *** |
|  | NRP1 | 0.329 | 0.549 | 0.526 | 0.356 | 0.515 | 0.584 | 0.73 | 0.329 | *** |
|  | ITGAX | 0.803 | 0.804 | 0.884 | 0.798 | 0.697 | 0.806 | 0.82 | 0.807 | *** |
| Th1 | TBX21 | 0.641 | 0.775 | 0.736 | 0.741 | 0.867 | 0.756 | 0.64 | 0.714 | *** |
|  | STAT4 | 0.585 | 0.721 | 0.662 | 0.724 | 0.801 | 0.787 | 0.684 | 0.588 | *** |
|  | STAT1 | 0.52 | 0.547 | 0.338 | 0.662 | 0.677 | 0.459 | 0.741 | 0.398 | *** |
|  | TNF | 0.516 | 0.338 | 0.363 | 0.559 | 0.69 | 0.327 | 0.631 | 0.192 | *** |
|  | IFNG | 0.461 | 0.541 | 0.531 | 0.509 | 0.757 | 0.489 | 0.684 | 0.572 | *** |
| Th1-like | HAVCR2 | 0.887 | 0.93 | 0.94 | 0.859 | 0.944 | 0.836 | 0.959 | 0.902 | *** |
|  | CXCR3 | 0.625 | 0.848 | 0.73 | 0.757 | 0.851 | 0.672 | 0.636 | 0.772 | *** |
|  | BHLHE40 | 0.169 | 0.102 | 0.308 | 0.46 | 0.03 | 0.134 | 0.401 | 0.045 | *** |
|  | CD4 | 0.946 | 0.953 | 0.908 | 0.936 | 0.935 | 0.914 | 0.911 | 0,892 | *** |
| Th2 | STAT6 | 0.252 | 0.138 | 0.263 | 0.444 | 0.092 | 0.317 | 0.265 | 0.144 | *** |
|  | STAT5A | 0.785 | 0.716 | 0.482 | 0.758 | 0.293 | 0.677 | 0.523 | 0.509 | *** |
| Treg | FOXP3 | 0.699 | 0.799 | 0.664 | 0.712 | 0.769 | 0.743 | 0.796 | 0.657 | *** |
|  | CCR8 | 0.74 | 0.807 | 0.551 | 0.74 | 0.813 | 0.77 | 0.684 | 0.508 | *** |
|  | TGFB1 | 0.553 | 0.201 | 0.708 | 0.707 | 0.545 | 0.533 | 0.328 | 0.391 | *** |
| Effector Treg T-cell | TNFRSF9 | 0.639 | 0.771 | 0.635 | 0.744 | 0.877 | 0.744 | 0.869 | 0.709 | *** |
|  | FGFBP2 | 0.256 | 0.005 | 0.085 | 0.395 | -0.06 | 0.378 | -0.077 | 0.091 | *** |
|  | FCGR3A | 0.802 | 0.864 | 0.897 | 0.775 | 0.845 | 0.683 | 0.824 | 0.714 | *** |
| Effector T-cell | CCR7 | 0.661 | 0.755 | 0.674 | 0.758 | 0.775 | 0.755 | 0.842 | 0,688 | *** |
|  | SELL | 0.704 | 0.737 | 0.615 | 0.817 | 0.817 | 0.795 | 0.741 | 0.674 | *** |
| Naïve T-cell | GZMK | 0.672 | 0.798 | 0.703 | 0.726 | 0.878 | 0.787 | 0.818 | 0.704 | *** |
|  | CD69 | 0.664 | 0.742 | 0.7 | 0.733 | 0.827 | 0.8 | 0.815 | 0.462 | *** |
| Effector memory T-cell | CXCR6 | 0.686 | 0.83 | 0.738 | 0.763 | 0.862 | 0.744 | 0.826 | 0.772 | *** |
|  | MYADM | 0.379 | 0.455 | 0.152 | 0.492 | 0.127 | 0.242 | 0.182 | 0.197 | *** |
| Resident memory T-cell | IL7R | 0.786 | 0.697 | 0.706 | 0.804 | 0.809 | 0.779 | 0.829 | 0.592 | *** |
|  | HAVCR2 | 0.887 | 0.93 | 0.94 | 0.859 | 0.944 | 0.836 | 0.959 | 0.902 | *** |
|  | LAG3 | 0.495 | 0.668 | 0.591 | 0.49 | 0.807 | 0.609 | 0.733 | 0.594 | *** |
|  | CXCL13 | 0.469 | 0.571 | 0.544 | 0.509 | 0.793 | 0.633 | 0.745 | 0.548 | *** |
| memory T-cell | LAYN | 0.4 | 0.05 | 0.066 | 0.61 | 0.441 | 0.533 | 0.031 | 0.284 | *** |

**Supplementary Table 3. The correlation between the UBE2C expression and drug sensitivity in diverse cancer cells lines analysis by the GDSC database.**

| symbol | drug | cor | fdr |
| --- | --- | --- | --- |
| UBE2C | NPK76-II-72-1 | 0.271889 | 0 |
| UBE2C | Trametinib | -0.44363 | 5.46E-43 |
| UBE2C | RDEA119 | -0.41227 | 2.62E-38 |
| UBE2C | selumetinib | -0.36725 | 7.97E-30 |
| UBE2C | 17-AAG | -0.35624 | 2.1E-25 |
| UBE2C | PD-0325901 | -0.34308 | 2.72E-22 |
| UBE2C | Docetaxel | -0.32626 | 1.03E-20 |
| UBE2C | (5Z)-7-Oxozeaenol | -0.30671 | 4.05E-19 |
| UBE2C | CI-1040 | -0.32111 | 7.59E-19 |
| UBE2C | Bleomycin (50 uM) | -0.29134 | 6.66E-18 |
| UBE2C | Vorinostat | 0.260879 | 1.12E-13 |
| UBE2C | WZ3105 | 0.24739 | 2.93E-13 |
| UBE2C | GSK690693 | 0.246988 | 6.18E-13 |
| UBE2C | YM201636 | 0.247249 | 6.59E-13 |
| UBE2C | KIN001-102 | 0.239324 | 1.81E-12 |
| UBE2C | AR-42 | 0.238137 | 3.39E-12 |
| UBE2C | Dabrafenib | -0.24968 | 3.74E-12 |
| UBE2C | QL-X-138 | 0.229793 | 2.89E-11 |
| UBE2C | PHA-793887 | 0.220249 | 8.22E-11 |
| UBE2C | GSK1070916 | 0.220846 | 1.92E-10 |
| UBE2C | BX-912 | 0.21636 | 2.08E-10 |
| UBE2C | TAK-715 | 0.218174 | 2.76E-10 |
| UBE2C | Tubastatin A | 0.213248 | 4.71E-10 |

**Supplementary Table 4. The correlation between the UBE2C expression and drug sensitivity in diverse cancer cells lines analysis by the CTRP database.**

| symbol | drug | cor | fdr |
| --- | --- | --- | --- |
| UBE2C | panobinostat | 0.351418 | 7.75E-23 |
| UBE2C | ciclopirox | 0.338518 | 3.38E-21 |
| UBE2C | SR-II-138A | 0.330798 | 1.15E-20 |
| UBE2C | BI-2536 | 0.329612 | 1.74E-20 |
| UBE2C | PX-12 | 0.336393 | 2.38E-20 |
| UBE2C | vorinostat | 0.332368 | 7.28E-20 |
| UBE2C | PL-DI | 0.325621 | 5.57E-19 |
| UBE2C | cerulenin | 0.325325 | 7.9E-19 |
| UBE2C | CR-1-31B | 0.315541 | 1.68E-18 |
| UBE2C | PAC-1 | 0.328769 | 1.88E-18 |
| UBE2C | BRD-K34222889 | 0.314814 | 4.69E-18 |
| UBE2C | apicidin | 0.308239 | 1.24E-17 |
| UBE2C | ceranib-2 | 0.307323 | 1.39E-17 |
| UBE2C | SB-225002 | 0.305403 | 1.65E-17 |
| UBE2C | isoevodiamine | 0.30918 | 1.74E-17 |
| UBE2C | piperlongumine | 0.306936 | 2.39E-17 |
| UBE2C | PRIMA-1 | 0.316185 | 3.49E-17 |
| UBE2C | neuronal differentiation inducer III | 0.307701 | 5.48E-17 |
| UBE2C | vincristine | 0.296633 | 5.99E-17 |
| UBE2C | ISOX | 0.30394 | 9.99E-17 |
| UBE2C | JQ-1 | 0.300766 | 1.17E-16 |
| UBE2C | belinostat | 0.42518 | 1.21E-16 |
| UBE2C | SB-743921 | 0.295033 | 1.83E-16 |
| UBE2C | ML311 | 0.296057 | 2.16E-16 |
| UBE2C | phloretin | 0.30454 | 3.07E-16 |
| UBE2C | cytarabine hydrochloride | 0.29477 | 3.98E-16 |
| UBE2C | entinostat | 0.299403 | 4.92E-16 |
| UBE2C | YK 4-279 | 0.292884 | 7.28E-16 |
| UBE2C | triazolothiadiazine | 0.288152 | 1.01E-15 |
| UBE2C | tacedinaline | 0.346713 | 1.48E-15 |
| UBE2C | LY-2183240 | 0.28893 | 1.87E-15 |
| UBE2C | GW-405833 | 0.29287 | 2.1E-15 |
| UBE2C | serdemetan | 0.30191 | 2.65E-15 |
| UBE2C | pifithrin-mu | 0.294886 | 3.52E-15 |
| UBE2C | Compound 23 citrate | 0.294992 | 3.81E-15 |
| UBE2C | narciclasine | 0.290785 | 3.86E-15 |
| UBE2C | parbendazole | 0.281891 | 4.54E-15 |
| UBE2C | LRRK2-IN-1 | 0.336723 | 7.32E-15 |
| UBE2C | mitomycin | 0.285279 | 8.41E-15 |
| UBE2C | BRD-K70511574 | 0.284332 | 8.95E-15 |
| UBE2C | PHA-793887 | 0.280786 | 1.09E-14 |
| UBE2C | BRD-K35604418 | 0.291358 | 1.13E-14 |
| UBE2C | indisulam | 0.29068 | 1.24E-14 |
| UBE2C | linifanib | 0.284542 | 1.4E-14 |
| UBE2C | BRD-K66453893 | 0.280692 | 2.03E-14 |
| UBE2C | BRD-K80183349 | 0.28481 | 3.02E-14 |
| UBE2C | GSK461364 | 0.278277 | 3.73E-14 |
| UBE2C | etoposide | 0.275285 | 4.64E-14 |
| UBE2C | MK-1775 | 0.280626 | 4.77E-14 |
| UBE2C | CHM-1 | 0.274049 | 6.22E-14 |
| UBE2C | doxorubicin | 0.264884 | 2.01E-13 |
| UBE2C | decitabine | 0.265863 | 2.61E-13 |
| UBE2C | FQI-2 | 0.26548 | 2.75E-13 |
| UBE2C | valdecoxib | 0.280221 | 3.02E-13 |
| UBE2C | KX2-391 | 0.267376 | 4.46E-13 |
| UBE2C | Ko-143 | 0.274669 | 5E-13 |
| UBE2C | sirolimus | 0.271831 | 5.13E-13 |
| UBE2C | nakiterpiosin | 0.265252 | 6.42E-13 |
| UBE2C | CD-437 | 0.261051 | 1.44E-12 |
| UBE2C | I-BET151 | 0.257348 | 1.54E-12 |
| UBE2C | BRD-K26531177 | 0.274269 | 1.58E-12 |
| UBE2C | necrosulfonamide | 0.31483 | 1.67E-12 |
| UBE2C | BRD-K41597374 | 0.267391 | 1.81E-12 |
| UBE2C | paclitaxel | 0.259976 | 2.43E-12 |
| UBE2C | TW-37 | 0.270572 | 2.45E-12 |
| UBE2C | rigosertib | 0.261223 | 3.27E-12 |
| UBE2C | gossypol | 0.267674 | 3.37E-12 |
| UBE2C | topotecan | 0.251786 | 4.05E-12 |
| UBE2C | gemcitabine | 0.265845 | 4.37E-12 |
| UBE2C | StemRegenin 1 | 0.263705 | 4.7E-12 |
| UBE2C | BRD-K88742110 | 0.270912 | 5.69E-12 |
| UBE2C | manumycin A | 0.248963 | 1.54E-11 |
| UBE2C | KU-60019 | 0.252489 | 1.85E-11 |
| UBE2C | SCH-79797 | 0.248855 | 2.21E-11 |
| UBE2C | BIX-01294 | 0.249687 | 2.42E-11 |
| UBE2C | BRD-A86708339 | 0.367497 | 2.86E-11 |
| UBE2C | tivantinib | 0.353077 | 2.91E-11 |
| UBE2C | Merck60 | 0.244175 | 3.04E-11 |
| UBE2C | BRD6340 | 0.254894 | 3.13E-11 |
| UBE2C | KPT185 | 0.284665 | 3.41E-11 |
| UBE2C | BRD-K51490254 | 0.267555 | 4.37E-11 |
| UBE2C | KHS101 | 0.261545 | 4.49E-11 |
| UBE2C | BMS-345541 | 0.247608 | 6.52E-11 |
| UBE2C | fingolimod | 0.247638 | 6.69E-11 |
| UBE2C | bardoxolone methyl | 0.260675 | 7.56E-11 |
| UBE2C | alisertib | 0.242072 | 7.86E-11 |
| UBE2C | BRD-K66532283 | 0.250582 | 7.87E-11 |
| UBE2C | NSC48300 | 0.242046 | 8.17E-11 |
| UBE2C | masitinib | 0.253427 | 8.29E-11 |
| UBE2C | omacetaxine mepesuccinate | 0.281537 | 1.08E-10 |
| UBE2C | GW-843682X | 0.247037 | 1.78E-10 |
| UBE2C | Compound 7d-cis | 0.259571 | 1.87E-10 |
| UBE2C | COL-3 | 0.278687 | 1.9E-10 |
| UBE2C | PF-184 | 0.240435 | 2.02E-10 |
| UBE2C | NSC632839 | 0.233931 | 2.87E-10 |
| UBE2C | curcumin | 0.234006 | 3.54E-10 |
| UBE2C | BRD-K92856060 | 0.244052 | 3.98E-10 |
| UBE2C | BRD-K61166597 | 0.23872 | 5.63E-10 |
| UBE2C | sotrastaurin | 0.275211 | 5.88E-10 |
| UBE2C | B02 | 0.236796 | 8.94E-10 |
| UBE2C | BRD-A94377914 | 0.316411 | 1.25E-09 |
| UBE2C | tipifarnib-P1 | 0.231587 | 1.26E-09 |
| UBE2C | MST-312 | 0.228141 | 1.31E-09 |
| UBE2C | linsitinib | 0.24183 | 1.36E-09 |
| UBE2C | RITA | 0.235533 | 1.52E-09 |
| UBE2C | axitinib | 0.224304 | 1.73E-09 |
| UBE2C | crizotinib | 0.225332 | 1.88E-09 |
| UBE2C | cucurbitacin I | 0.230962 | 1.96E-09 |
| UBE2C | PDMP | 0.239031 | 2.01E-09 |
| UBE2C | GSK525762A | 0.221159 | 2.07E-09 |
| UBE2C | ML029 | 0.243535 | 2.28E-09 |
| UBE2C | barasertib | 0.223606 | 2.67E-09 |
| UBE2C | ouabain | 0.220101 | 2.86E-09 |
| UBE2C | NVP-231 | 0.220713 | 3.52E-09 |
| UBE2C | WP1130 | 0.225668 | 4.46E-09 |
| UBE2C | methylstat | 0.264044 | 5.3E-09 |
| UBE2C | oligomycin A | 0.223887 | 5.59E-09 |
| UBE2C | BRD1812 | 0.222927 | 5.82E-09 |
| UBE2C | OSI-930 | 0.242794 | 6E-09 |
| UBE2C | teniposide | 0.29702 | 7.25E-09 |
| UBE2C | dinaciclib | 0.310943 | 7.44E-09 |
